# Hi-C Metagenomics in the ICU: Exploring Clinically Relevant Features of Gut Microbiome in Chronically Critically Ill Patients

**DOI:** 10.3389/fmicb.2021.770323

**Published:** 2022-02-03

**Authors:** Valeriia Ivanova, Ekaterina Chernevskaya, Petr Vasiluev, Artem Ivanov, Ivan Tolstoganov, Daria Shafranskaya, Vladimir Ulyantsev, Anton Korobeynikov, Sergey V. Razin, Natalia Beloborodova, Sergey V. Ulianov, Alexander Tyakht

**Affiliations:** ^1^Institute of Gene Biology Russian Academy of Sciences, Moscow, Russia; ^2^Federal Research and Clinical Center of Intensive Care Medicine and Rehabilitology, Moscow, Russia; ^3^Research Centre for Medical Genetics, Moscow, Russia; ^4^Computer Technologies Laboratory, ITMO University, Saint Petersburg, Russia; ^5^Center for Algorithmic Biotechnologies, Saint Petersburg State University, Saint Petersburg, Russia; ^6^Faculty of Biology, Lomonosov Moscow State University, Moscow, Russia; ^7^Center for Precision Genome Editing and Genetic Technologies for Biomedicine, Institute of Gene Biology Russian Academy of Sciences, Moscow, Russia

**Keywords:** Hi-C metagenomics, critical care, antibiotic resistance, plasmids, *Klebsiella*, metagenome-assembled genome, binning, gut microbiome

## Abstract

Gut microbiome in critically ill patients shows profound dysbiosis. The most vulnerable is the subgroup of chronically critically ill (CCI) patients – those suffering from long-term dependence on support systems in intensive care units. It is important to investigate their microbiome as a potential reservoir of opportunistic taxa causing co-infections and a morbidity factor. We explored dynamics of microbiome composition in the CCI patients by combining “shotgun” metagenomics with chromosome conformation capture (Hi-C). Stool samples were collected at 2 time points from 2 patients with severe brain injury with different outcomes within a 1–2-week interval. The metagenome-assembled genomes (MAGs) were reconstructed based on the Hi-C data using a novel hicSPAdes method (along with the bin3c method for comparison), as well as independently of the Hi-C using MetaBAT2. The resistomes of the samples were derived using a novel assembly graph-based approach. Links of bacteria to antibiotic resistance genes, plasmids and viruses were analyzed using Hi-C-based networks. The gut community structure was enriched in opportunistic microorganisms. The binning using hicSPAdes was superior to the conventional WGS-based binning as well as to the bin3c in terms of the number, completeness and contamination of the reconstructed MAGs. Using *Klebsiella pneumoniae* as an example, we showed how chromosome conformation capture can aid comparative genomic analysis of clinically important pathogens. Diverse associations of resistome with antimicrobial therapy from the level of assembly graphs to gene content were discovered. Analysis of Hi-C networks suggested multiple “host-plasmid” and “host-phage” links. Hi-C metagenomics is a promising technique for investigating clinical microbiome samples. It provides a community composition profile with increased details on bacterial gene content and mobile genetic elements compared to conventional metagenomics. The ability of Hi-C binning to encompass the MAG’s plasmid content facilitates metagenomic evaluation of virulence and drug resistance dynamics in clinically relevant opportunistic pathogens. These findings will help to identify the targets for developing cost-effective and rapid tests for assessing microbiome-related health risks.

## Introduction

The number of patients with long-term dependence on support systems in the ICU is growing with the improvement in the quality of medical care. Chronically critically ill (CCI) patients are a heterogeneous group of patients after long stay in ICU characterized by the unique physiology including hormonal and metabolic changes with hypermetabolic and hypercatabolic state (Nelson et al., 2010; Parfenov et al., 2020), cognitive impairment, myopathy, inflammation (Cox, 2012) and increased susceptibility to infection (Nierman and Nelson, 2002). Gastrointestinal tract diseases can be observed in most such patients due to impaired swallowing, insufficient physical activity, horizontal position, feeding through a gastric tube or stoma, fluid and electrolyte disorders. The profound dysbiosis of the gut microbiome has a great influence on the gastrointestinal tract (Foster, 2016), leading to inflammation and tissue injury. These changes form a vicious pathological circle that prevents recovery. In addition, these patients receive multiple classes of antibiotics during hospitalization to treat ventilator-associated pneumonia, bloodstream infections caused by use of a central venous catheter, as well as the urinary tract infections, thus contributing toward the evolution of their microbiome into a reservoir of virulent multidrug-resistant nosocomial pathogens. Previously, we characterized the taxonomic composition of microbiome in CCI patients using 16S rRNA sequencing (Chernevskaya et al., 2020). The observed dysbiosis was linked to prognosis and reflected in the altered spectrum of microbially produced phenolic metabolites in blood and stool (Chernevskaya et al., 2021). It suggested the necessity of further investigation of the genetic potential of the microbial species abundant in CCI – including mobile genetic elements – using more powerful approaches like “shotgun” metagenomics.

Recently, conventional metagenomics have been combined with chromosome conformation capture techniques like Hi-C and 3C-seq to enable a deeper exploration of complex microbial communities. Besides the environmental microbiome (Baudry et al., 2019; Stalder et al., 2019), such techniques have been applied to mammal host-associated communities (Marbouty et al., 2017; Stewart et al., 2018; Bickhart et al., 2022). In the human microbiome field, all but one Hi-C metagenomic survey (Kent et al., 2020) performed to date investigated the gut community of healthy subjects (Press et al., 2017; DeMaere et al., 2020; Marbouty et al., 2021). In these studies, the overimposement of the paired Hi-C reads reflecting the information about chromosome spatial proximity onto the metagenomic assembly allowed better binning of the contigs into metagenome-assembled genomes (MAGs). Few software tools developed for Hi-C genome deconvolution have been published (Baudry et al., 2019; DeMaere and Darling, 2019).

By exploiting the fact that the chromosome interaction signal is higher across the genomic sequences present in the same microbial cell, it was possible to suggest specific “phage-microbe” and “plasmid-microbe” links. The mobile genetic elements are responsible for the horizontal gene transfer (HGT) that is of high biomedical relevance due to the transmission of antibiotic resistance and virulence factors genes. Therefore, application of Hi-C metagenomics to the human microbiome in the clinical context is promising for assessing microbiome-associated health risks in patients, especially in the immunocompromised ones. The only study of the alterations of gut microbiome in disease focused on the neutropenic patients undergoing hematopoietic stem cell transplantation (Kent et al., 2020).

In our study, we established an experimental and bioinformatic pipeline for Hi-C metagenomic analysis involving novel algorithms and applied it to explore the functional dynamics of gut dysbiosis in a pilot set of samples from CCI patients, with particular focus on evaluating such essential advantages of the technique compared to the conventional WGS as improved reconstruction of microbial genomes and a possibility to link antibiotic resistance genes, plasmids and viruses to their hosts.

## Materials and Methods

### Study Design

**FIGURE 1:**
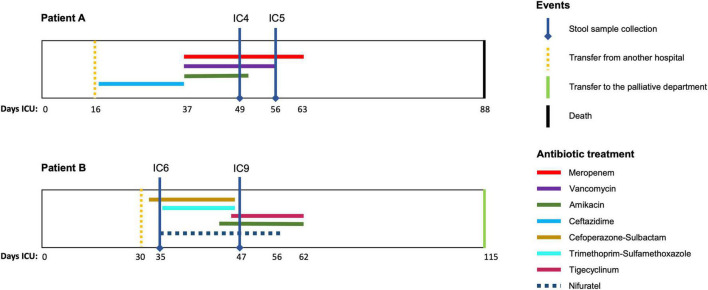
Antimicrobial therapy timelines for the patients. The time courses of the two patients **(A,B)** included in the study are shown along with the periods (days) of antimicrobial drug administration (colored lines). Vertical lines indicate the key time points for the patients.

This prospective observational study was performed in the Department of Intensive Care at the Federal Research and Clinical Center of Intensive Care Medicine and Rehabilitology, Moscow, Russian Federation. The stool samples were collected from two CCI patients: patient A – a 75 year old female after an intracerebral hemorrhage and patient B – a 74 year old male after an ischemic stroke. Both patients were on prolonged mechanical ventilation and enteral tube feeding (high calorie, low-residue) and received antibiotics (Figure 1). At the first time point, each had a suspected bacterial infection (pathogens were isolated from trachea, chest CT scans showed pneumonia). The second time point at which the stool was collected was day 7 for patient A (with negative clinical dynamics) and day 14 – for patient B (positive clinical dynamics). The detailed clinical data is provided in the Additional File 1: Supplementary Table 1.

### Sample Preparation and Sequencing

The WGS (metagenomic) libraries were prepared using the NEBNext Ultra II FS DNA Library Prep Kit for Illumina exactly according to the manufacturer’s instructions. Total genomic DNA was isolated as follows. Cell material was incubated in a 1× TE buffer at 65°C for 14–16 h in the presence of proteinase K (1 μg/μl) and 0.5% of SDS. DNA was then purified by single phenol-chloroform extraction followed by ethanol precipitation with 20 μg/ml glycogen (Thermo Fisher Scientific) as the co-precipitator. After precipitation, the pellets were dissolved in 50 μl 10 mM Tris–HCl pH 8.0. To remove residual RNA, samples were treated with 25 μg of RNase A (Thermo Fisher Scientific) for 45 min at 37°C. To remove residual salts and DTT, the DNA was additionally purified using Agencourt AMPure XP beads (Beckman Coulter). Then 20–100 ng of the purified DNA was used for WGS library preparation.

The Hi-C libraries were prepared as described below, in two replicates per sample. The sample was resuspended in saline solution (NaCl 0.9%), homogenized and centrifuged at 200 × *g* for 5 min, to precipitate the debris. Supernatant was removed in a separate tube and centrifuged at 10,000 × *g* for 5 min. The pellet was homogenized for 40 s in tubes containing Lysing Matrix A (MP Biomedicals) and a 6 mm ceramic sphere using an MP Biomedicals FastPrep-24 instrument at 6 m/s, then was resuspended in 1 ml of saline solution and centrifuged at 10,000 × *g* for 5 min 3 more times. Then the pellet was resuspended in 1 ml of fixing solution (NaCl 0.9%, formaldehyde 3%) and incubated for 20 min at 22°C with tube inversion every 2 min. The reaction was stopped by the addition of 2 M glycine to give a final concentration of 125 mM. Cells were centrifuged (17,000 × *g*, 10 min, 4°C), resuspended in 50 μl of 1 × PBS, snap-frozen in liquid nitrogen and stored at −80°C. Defrozen cells were mechanically disrupted using a Dounce homogenizer and additionally lysed in 1 ml isotonic buffer [50 mM Tris–HCl pH 8.0, 150 mM NaCl, 0.5% (v/v) NP-40 substitute (Fluka), 1% (v/v) Triton-X100 (Sigma), 1 × Halt Protease Inhibitor Cocktail (Thermo Fisher Scientific)] on ice for 15 min. Cells were centrifuged at 20,000 × *g* for 5 min at 4°C, resuspended in 200 μl of 1 × NEBuffer 2 (NEB), and pelleted again. The pellet was resuspended in 200 μl of 0.3% SDS in 1 × NEBuffer 2 and incubated at 37°C for 1 h. Then, cells were centrifuged at 20,000 × *g* for 5 min at 4°C, washed with 200 μl of 1 × NEBuffer 2 and resuspended in 1 × CutSmart buffer (NEB) supplemented with 1% of Triton X-100 (Sigma). 100 U of *Hpa*II enzyme (NEB) were added, and the DNA was digested overnight (14–16 h) at 37°C with shaking (1,400 rpm). On the following day, additional 100 U of *Hpa*II enzyme were added, and the cells were incubated for an additional 2 h. *Hpa*II was then inactivated by incubation at 65°C for 20 min. After *Hpa*II inactivation, the cells were harvested for 10 min at 20,000 × *g*, washed with 300 μl of 1 × T4 DNA ligase buffer (Fermentas), and resuspended in 300 μl of 1 × T4 DNA ligase buffer. Cohesive DNA ends were ligated in the presence of 75 U of T4 DNA ligase (Fermentas) at 16°C for 4 h. The cross-links were reversed by overnight incubation at 65°C in the presence of proteinase K (1 μg/μl) (Sigma) and 0.5% of SDS. After cross-link reversal, the DNA was purified by single phenol-chloroform extraction followed by ethanol precipitation with 20 μg/ml glycogen (Thermo Fisher Scientific) as the co-precipitator. After precipitation, the pellets were dissolved in 100 μl 10 mM Tris–HCl pH 8.0. To remove residual RNA, samples were treated with 50 μg of RNase A (Thermo Fisher Scientific) for 45 min at 37°C. To remove residual salts and DTT, the DNA was additionally purified using Agencourt AMPure XP beads (Beckman Coulter). The DNA was then dissolved in 500 μl of sonication buffer [50 mM Tris–HCl (pH 8.0), 10 mM EDTA, 0.1% SDS] and sheared to a size of approximately 100–1,000 bp using a VirSonic 100 (VerTis). The samples were concentrated (and simultaneously purified) using AMICON Ultra Centrifugal Filter Units to a total volume of approximately 50 μl. The DNA ends were repaired by adding 62.5 μl MQ water, 14 μl of 10 × T4 DNA ligase reaction buffer (Fermentas), 3.5 μl of 10 mM dNTP mix (Fermentas), 5 μl of 3 U/μl T4 DNA polymerase (NEB), 5 μl of 10 U/μl T4 polynucleotide kinase (NEB), 1 μl of 5 U/μl Klenow DNA polymerase (NEB), and then incubating at 20°C for 30 min. The DNA was purified with Agencourt AMPure XP beads and eluted with 127 μl of 10 mM Tris–HCl (pH 8.0). To perform an A-tailing reaction, the DNA samples were supplemented with 15 μl 10 × NEBuffer 2, 3 μl of 10 mM dATP, and 4.5 μl of 5 U/μl Klenow (exo-) (NEB). The reactions were carried out for 30 min at 37°C in a PCR machine, and the enzyme was then heat-inactivated by incubation at 65°C for 20 min. The DNA was purified using Agencourt AMPure XP beads and eluted with 100 μl of 10 mM Tris–HCl (pH 8.0). Illumina TruSeq adapters were ligated by adding 12 μl 10 × T4 DNA ligase reaction buffer (Fermentas), 6 μl of Illumina TruSeq adapters and 2 μl of 5 U/μl T4 DNA ligase (Fermentas). Adapter ligation was performed at 22°C overnight. DNA was then purified with Agencourt AMPure XP beads and eluted with 30 μl of 10 mM Tris–HCl (pH 8.0). Test PCR reactions containing 5 μl of the samples were performed to determine the optimal number of PCR cycles required to generate sufficient PCR products for sequencing. The PCR reactions were performed using KAPA High Fidelity DNA Polymerase (KAPA) and Illumina PE1.0 and PE2.0 PCR primers (10 pmol each). The temperature profile was 5 min at 98°C, followed by 6, 9, 12, 15, and 18 cycles of 20 s at 98°C, 15 s at 65°C, and 20 s at 72°C. The PCR reactions were separated on a 2% agarose gel containing ethidium bromide, and the number of PCR cycles necessary to obtain a sufficient amount of DNA was determined based on the visual inspection of gels (typically 10–12 cycles). Four preparative PCR reactions were performed for each sample. The PCR mixtures were combined, and the DNA was purified using Agencourt AMPure XP beads and eluted with 50 μl of 10 mM Tris–HCl (pH 8.0).

The sequencing of the WGS and Hi-C libraries was performed on the Illumina HiSeq platform in 2 × 150 bp reads format.

Additionally, the abundance of selected gut taxa was measured using multiplex real-time PCR with fluorescent detection with the Colonoflor-16 kit (Alfalab, Russia).

### Analysis of the WGS and Hi-C Data

We established the analytical pipeline for combined analysis of the “shotgun” readsets and their chromosome conformation capture counterparts; the workflow diagram is outlined in Additional File 1: Supplementary Figure 1. At the WGS prefiltering step, read merging and adapter removal was performed by bbmerge v.37.62 (with default parameters and k = 61, adapter = default). The WGS reads were assembled using SPADES v.3.15 (Prjibelski et al., 2020) in “meta” mode. Replicates of Hi-C libraries were pooled before processing and then filtered with BBMap (bbduk). For each sample, the binning of contigs was performed without the Hi-C data – into WGS-MAGs, *via* MetaBat 2 (Kang et al., 2019) – and using Hi-C data – into Hi-C MAGs, *via* the novel hicSPAdes algorithm.^1^ As an additional option, the Hi-C MAGs have been produced using the previously published bin3c algorithm (DeMaere and Darling, 2019).

Taxonomic profiling of metagenomic reads was performed using MetaPhlAn2 (Truong et al., 2015) and MiCoP (LaPierre et al., 2019). The MAG quality was evaluated using CheckM (Parks et al., 2015). The taxonomy of each MAG was inferred using GTDB-Tk (Chaumeil et al., 2019). Circular packing plots of MAGs were generated using packcircles R package.^2^ The quality of Hi-C libraries was evaluated with qc3C (DeMaere and Darling, 2021). Comparative genome analysis and visualization of the MAGs and published reference genomes was performed using anvi’o pipeline (Eren et al., 2015) in the pangenomic workflow including: ‘anvi-script-FASTA-to-contigs-db’ script – for contigs database construction for each MAG, ‘anvi-run-ncbi-cogs’ – for annotating the genes according to the NCBI Clusters of Orthologous Groups database, ‘anvi-gen-genomes-storage’ – for creating genome storage, ‘anvi-pan-genome’ – for generating pan database (particularly, search for amino acid sequence similarity with blastp and gene clusters identification), ‘anvi-display-pan’ – for visualization in the interactive interface, and ‘anvi-summarize’ – for generating static summary; the genes were predicted using Prodigal (Hyatt et al., 2010). Tree was constructed based on SCGs by FastTree and MAFFT, using ‘anvi-gen-phylogenomic-tree’ script. Genes encoding virulence factors were identified using the VFanalyzer tool with the VFDB database (Liu et al., 2019). UpSet plots were generated using the UpsetR package (Conway et al., 2017).

For evaluating the number of plasmid-like contigs in the assembly and the MAGs, PlasFlow (Krawczyk et al., 2018) was used (followed by blastn to the NCBI plasmid database for taxonomic validation). Prophage sequences in contigs were identified using PHASTER (Arndt et al., 2019). Search and annotation of antibiotic resistance genes (ARGs) in the MAGs and contigs were conducted with RGI (Resistance Gene Identifier)^3^ using the CARD database (Alcock et al., 2020). The whole-metagenome resistome was assessed using the GraphAMR pipeline (Shafranskaya et al., 2021). GraphAMR overcomes the limitations of fragmented contigs in metagenomic assemblies (due to interspecies repeats, horizontal gene transfer and other mechanisms) *via* aligning the ARG profile Hidden Markov Model directly to the assembly graph with subsequent dereplication and identification. This approach allows for accurate and comprehensive recovery of ARGs without the necessity of their assembly into a complete contig. The method is able to detect an ARG even if it is not assembled (and thus spans multiple contigs).

For the construction of Hi-C contig networks, the contigs > 1,000 bp were selected as vertices. The Hi-C reads were mapped to the contigs using bwa. For each contig pair, the number of Hi-C read pairs connecting them was normalized using HiCzin^4^ based on contigs’ coverage and length as well as intra-species contacts. Intra-species contacts were estimated as the contacts between contigs within high-quality MAGs (with completeness > 80% and contamination < 5%). Using the normalized weights, we constructed distributions of intra-MAG and inter-MAG contacts intensity for the high-quality MAGs, for each sample (Additional File 1: Supplementary Figure 2A). Based on these histograms, we visually determined a threshold value of 0.6 – as it fits well across the samples to remove most inter-MAG contacts while retaining most intra-MAG contacts. Only the Hi-C links with normalized weight above this threshold were used to construct the network edges. During the analysis of “microbe-phage” links, we considered a viral contig to be connected to a bacterial MAG if it had a link to at least one of the MAG’s contigs in the normalized interaction network with a weight above the threshold (0.6).

The classification of contigs as chromosomal/viral/plasmid was conducted using ViralVerify (Antipov et al., 2020) (with the -p flag used for the plasmid search). Draft taxonomic annotation of contigs was obtained using Kraken (Wood and Salzberg, 2014). The networks were visualized in Cytoscape (Su et al., 2014).

Taxonomic classification of the predicted viral contigs from assemblies was performed using DemoVir^5^ to the levels of order and family. In an analysis complementary to the Hi-C-based approach, prediction of associations between viral contigs and bacterial MAGs was performed using VirMatcher – based on viral sequence matches to host CRISPR-spacers, integrated prophages in host genomes, host tRNA genes, and host k-mer signatures calculated by WisH (Gregory et al., 2020). Only the matches with final score ≥ 3 (according to the guidelines provided in the software repository)^6^ were considered. Simulation of WGS reads (10 mln read pairs per sample) was performed using InSilicoSeq (Gourlé et al., 2019). For simulating Hi-C reads, the Sim3C tool (DeMaere and Darling, 2018) was used (5 mln read pairs per sample, read length 150 bp, *Hpa*II enzyme).

## Results

### Basic Analysis of Gut Community Structure: Pronounced Dysbiosis

The WGS sequencing of 2 pairs of stool samples produced 109–131 mln read pairs per sample. A preliminary taxonomic profiling of the patients’ gut metagenomes was performed using unique clade-specific gene markers (see section “Materials and Methods”). It revealed pronounced dysbiosis, particularly, with the levels of Proteobacteria 1–2 orders higher than observed from NGS microbiome surveys for the general Russian population (Tyakht et al., 2013; Klimenko et al., 2018; Volokh et al., 2019). The disruption of gut community structures has been confirmed *via* a complementary analysis using taxon-specific qPCR (Additional File 2: Supplementary Table 2). The decreased diversity was driven by *Bacteroidaceae* and various opportunist genera (*Klebsiella*, *Escherichia*, *Proteus*, *Bilophila*; see Additional File 1: Supplementary Figure 3). Besides the prokaryotes, there were fungal sequences normally not observed in healthy populations: the intracellular parasite *Enterocytozoon bieneusi* was omnipresent, with other detections including *Aspergillus niger* and *Candida glabrata* (Additional File 3: Supplementary Table 3).

### Hi-C Allows to Obtain Higher Quality Metagenome-Assembled Genomes Compared to WGS

**FIGURE 2:**
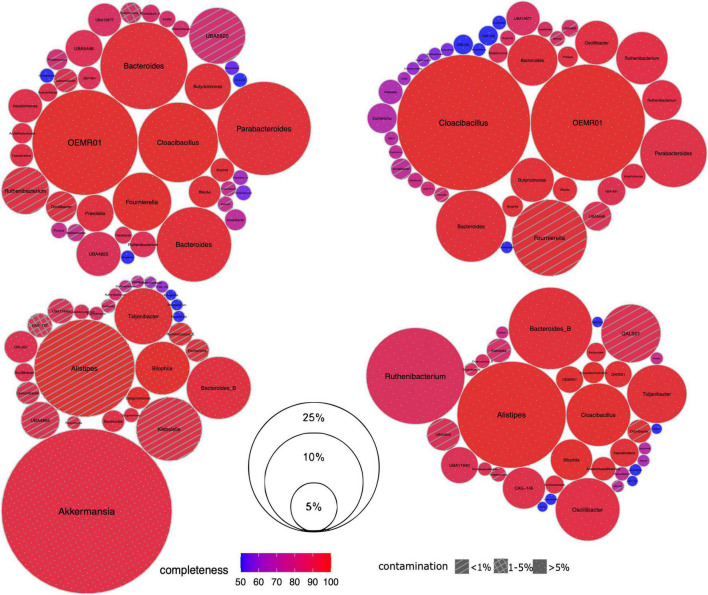
Taxonomic composition of gut microbiome in CCI patients. For each sample, its set of recovered Hi-C MAGs (*via* hicSPAdes) is visualized as circle packing. Each circle represents a MAG labeled with genus-level taxonomy; MAG relative abundance (normalized by the total length of its contigs) is shown as its diameter, completeness – as color, and contamination – as fill pattern. Sample IDs from left to right, top to bottom: IC4, IC5, IC6 and IC9.

Next we investigated the microbiome composition of the patients at a deeper level *via* the reconstruction of MAGs (Figure 2). For each sample, the assembly was of relatively good quality including 91,381–139,339 contigs > 200 bp long, with a maximum length of 523,891–783,225 bp and the N50 value of 4,196–9,150 bp. According to the qc3c analysis, the estimated fraction of Hi-C reads was 18.28 – 47.55%, suggesting overall proper ligation.

Two types of MAGs were reconstructed for each sample – one being Hi-C-agnostic (WGS-MAGs) and another one exploiting the Hi-C linkage information (Hi-C MAGs). The conventional WGS binning was conducted using MetaBat2 as a state-of-art WGS binning algorithm. The Hi-C binning was performed in 2 versions. The first one used hicSPAdes – a novel binning and binning improvement tool that simultaneously exploits the information from Hi-C-derived links and topology of the assembly graph to improve the completeness and purity of MAG bins; the second – performed for comparison purposes – was the existing bin3c algorithm (see Additional File 4: Supplementary Table 4 for summary statistics of libraries, assemblies and binnings).

**FIGURE 3:**
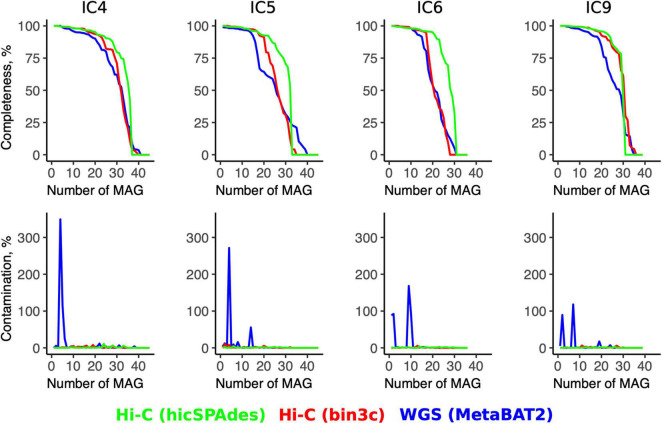
Quality metrics for the Hi-C- and WGS-MAGs. The line plots show the values of MAGs’ completeness (top row) and contamination (bottom row) for each sample (column-wise) according to the WGS binning and two versions of Hi-C binnings (shown in three colors). For each sample, the MAGs are sorted in the order of decreasing completeness.

Even when the Hi-C binning was performed using bin3c (Additional File 5: Supplementary Tables 5A–D), the number of produced high-quality MAGs (completeness > 80%, contamination < 5%) was higher than for the WGS (bin3c: 16–25 vs. MetaBAT2: 11–20) (Additional File 6: Supplementary Table 6). The contamination across the high-quality MAGs did not exceed 11% for Hi-C, while some WGS-MAGs had levels up to 300% or higher (Figure 3).

The superiority of the Hi-C approach was even more pronounced when the novel hicSPAdes was used for obtaining the Hi-C MAGs (23–27 high-quality MAGs and contamination < 7% for all MAGs) (Additional File 7: Supplementary Table 7). Compared to the WGS, the completeness was significantly higher for the Hi-C MAGs produced by hicSPAdes (while no significance was achieved in the case of bin3c; *t*-test for MAGs pooled across the 4 samples, *p* = 0 and 0.58, respectively).

High contamination levels close to multiples of 100% sporadically manifested by some WGS-MAGs were due to erroneous conglomeration of 2 or more genomes. The Hi-C approach allowed to resolve such cases. As an illustrative example of this effect, a *Dysgonomonas* WGS-MAG from the sample IC6 had a 88.5% contamination. It corresponded to two high-quality Hi-C MAGs (*via* hicSPAdes) – each classified at genus level as *Dysgonomonas* with contamination < 1% and completeness of 98.01–99.93% (Additional Files 6, 7: Supplementary Tables 6, 7). The two Hi-C counterparts of a *Bacteroides dorei* WGS-MAG (contamination: 92.2%, strain heterogeneity: 50.0%) were a *B. dorei* and *B. xylanisolvens*—with 0.38 and 0% contamination, respectively.

We analyzed the proportions of the assembly that were not binned into the MAGs. In terms of assembly length proportion, the Hi-C MAGs included 55.6–64.3% of the total contig length, while for the WGS, the sum was 54.9–63.4%. Considering a lower contamination in Hi-C MAGs, it suggests that they are generally more encompassing and provide more complete gene content for each member of the microbiome community, while balancing it with detailedness of species-level disentanglement.

As the set of abundant species was considerably overlapping between the timepoints, we also performed per-patient cross-assembly to assess how it improves the completeness of the reconstructed Hi-C MAGs (here we chose bin3c as an established binning algorithm to serve as a baseline). The results of the cross-assembly and comparison with the sample-wise Hi-C MAGs are shown in Additional File 5: Supplementary Tables 5E,F. In this analysis, we excluded the MAGs of low completeness (<10%) unclassified according to GTDBtk. The effect on MAG quality was ambiguous. For patient B, there were 16 (32.7%) cross-assembled MAGs that improved in quality (for some – dramatically) and 4 MAGs with novel taxonomy were obtained. For 20 MAGs, their completeness did not improve by remaining close to the maximum across the two time points; nine MAGs had their completeness decreased. For patient A, the respective numbers of Hi-C MAGs were: 25 (43.9%) – were improved, 10 novel, 11 – had similar completeness and 11 – decreased in completeness.

Noteworthy, the cross-assembly did not promote contamination for most Hi-C MAGs. Such an increase was observed for both patients: for patient A, only 4 of her MAGs with contamination < 5% received values > 5% in the cross-assembly (but remained below 15.2%); for patient B, there were two such cases (for them, contamination was < 11.5%).

### Comparative Genome Analysis for Major Opportunist Taxa Facilitated by Hi-C

We evaluated the advantages of Hi-C MAGs to explore the virulence and drug resistance potential of the opportunist taxa enriched in the CCI microbiome. For the proof-of-principle, we selected the *Klebsiella pneumoniae* (Kp) – the species abundant in all 4 samples and represented by a single Hi-C MAG in each of them. (The hicSPAdes version of Hi-C MAGs were used in all samples but IC4 – in the latter, hicSPAdes did not produce a Kp MAG during the binning so we used the bin3c version instead).

**FIGURE 4:**
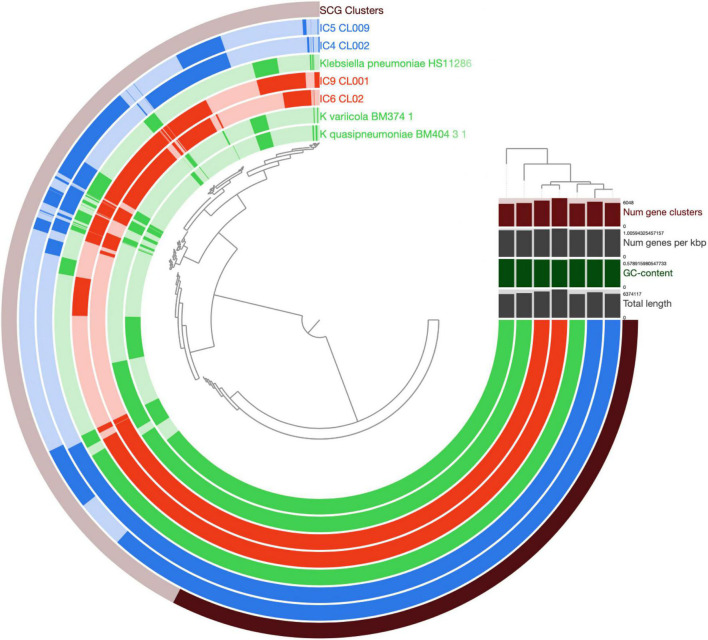
Comparison of *K. pneumoniae* MAGs across the patients and time points. The genomes of *K. pneumoniae* strain HS11286, *K. variicola* BM374-1 and *K. quasipneumoniae* BM404-3-1 were included as external references. The circular hierarchical clustering diagram was constructed using the anvi’o pipeline (see section “Materials and Methods”). Each concentric circle represents a genome, while each radial ray corresponds to a gene (gene orthology cluster). The outermost circle shows the SCG genes (in black). The genomes/circles were hierarchically clustered based on their SCG sequence similarity, while the genes – on their prevalence pattern across the genomes. For the circles, the Kp MAGs of patient A and B are shown in blue and red, respectively; the reference genomes – in green. Saturated colors correspond to present genes, while pale ones – to the absent ones. In the upper right part, the total number of gene clusters, the gene density (number of genes per Kbp of genome), GC content (%) and total length are provided for each genome/MAG.

**FIGURE 5:**
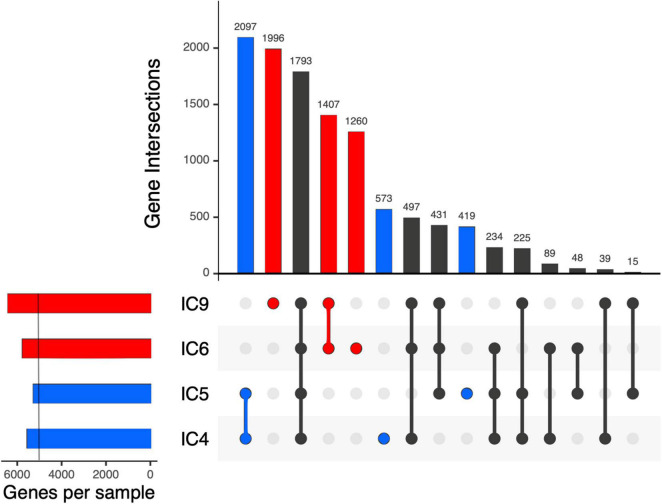
Intersection of the *K. pneumoniae* gene sets across the samples and patients using UpSet plots. Each vertical bar shows the number of genes common across the samples highlighted by dots below the bar. Subject-specific sets are shown in blue and red for patient A and B, respectively. On the left, a light vertical line denotes *n* = 5,000 genes.

After the gene prediction, the four MAGs were subject to clustering by their shared single-copy core genes (SCG) content similarity together with the reference genomes representative of the 3 known *K. pneumoniae* phylotypes (Figure 4). The results suggested that the CCI patients hosted *K. pneumoniae sensu stricto* (KpI phylotype), the one most commonly associated with human infection (Holt et al., 2015). The Kp MAGs clustered by subject; while the gene count was considerably lower for patient A than for B (5,547–5,262 vs. 5,759–6,403, according to anvi’o), the number of subject-wise genes persisting between the time points was, on the contrary, higher for A (2,097 vs. 1,407; Figure 5).

Evaluation of the Kp virulence potential from its Hi-C MAGs yielded 89–112 genes encoding virulence factors (VF; Additional File 8: Supplementary Table 8) suggesting these microbiomes host virulent Kp types. The VF lists included pili, fimbriae, efflux pumps, colibactin, capsule genes along with the RcsAB and RmpA systems regulating its production, the iron-scavenging siderophores salmochelin, aerobactin and yersiniabactin (the latter being the most common virulence factor associated with human *K. pneumoniae* infections); type VI secretion systems and the *rfb* locus responsible for lipopolysaccharide (LPS) biosynthesis. Allantoin utilization genes associated with liver hypervirulent strains were not detected in any of the MAGs. While the number of VF genes was close across all 4 samples, their proportion among all genes was higher for the samples from patient A (due to shorter Kp MAGs in both of them). Interestingly, only a few VF genes were subject-specific. For patient A, it was a type VI secretion system *tle1* phospholipase effector gene involved in bacterial competition. Another difference between the patients was in the set of fimbrial adherence determinants (likely acquired from *Salmonella*; all belonging to chaperone/usher fimbriae): in patient A, these were the genes of *stb* from *γ4* clade, while in patient B – of *ste* and *stf* from π clade (Dufresne et al., 2018).

Noteworthy, the WGS-MAGs contained fewer VF genes than the Hi-C MAGs (WGS: 66–101; Hi-C: 89–112); their proportion was also lower – for all samples but IC6.

### Plasmid Completeness of Metagenome-Assembled Genomes

**FIGURE 6:**
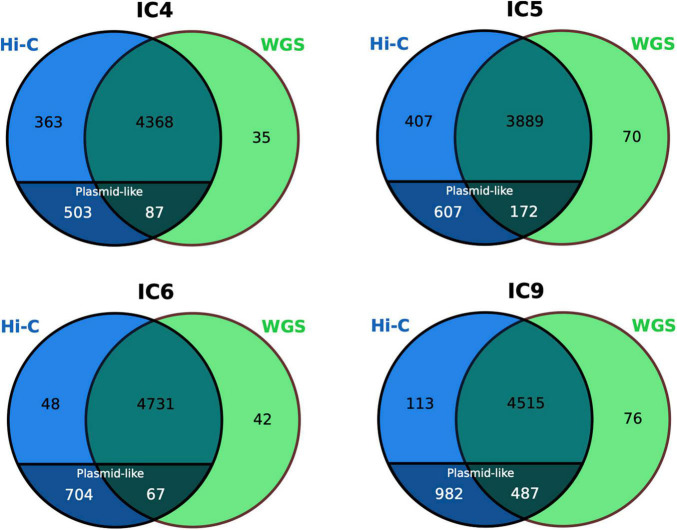
Comparison of the *K. pneumoniae* gene set between the WGS and Hi-C MAGs. The numbers for the plasmid-like contigs of Hi-C MAGs are particularly shown. The Venn diagrams are based on COG annotation.

Specifically in the case of *K. pneumoniae*, the conventional completeness and contamination metrics of its MAGs (as assessed *via* CheckM) were not considerably different between the WGS and Hi-C versions. The completeness was > 91% and contamination – < 3% in most cases. However, these metrics are not particularly focused on accounting for the accessory genes and plasmids. Plasmids are an important HGT channel responsible for dissemination of AR genes in microbial communities, particularly, within the human gut (McInnes et al., 2020). We evaluated whether the Hi-C MAGs portrayed a more complete gene and plasmid content of the species than the WGS (Figure 6).

Noteworthy, while only a handful of genes were unique to the WGS-MAGs (*n* = 35–76), the sets of Hi-C-unique genes were as large as *n* = 752–1,095. Decomposition of the latter showed that, although the Hi-C MAGs’ contamination was low (<2.5%), their total length was higher than of their WGS counterparts not just due to a higher chromosomal completeness, but also due to the inclusion of plasmid-like contigs – that were underrepresented in the WGS-MAGs (*n* ≤ 7 per each). The fact that such plasmid content is not mostly a false-positive inclusion from unrelated taxa is supported by the BLAST search against nr database showing that all classified contigs belonged to *Klebsiella* or related taxa (*Enterobacter*, *Salmonella*, *Escherichia*, *Raoultella*, *Pseudomonas*, *Citrobacter* or *Acinetobacter* – the Gammaproteobacteria order, mostly from *Enterobacteriaceae* family).

We evaluated how Hi-C data can help establish the links of plasmids to their bacterial hosts at the community level. Firstly, identification of potential plasmid-related contigs in the 4 metagenomes suggested an extensive presence of plasmids, with the Proteobacteria and *Enterococci* showing disproportionately high contribution to the plasmid pool compared to the commensal taxa (Additional File 1: Supplementary Figure 4). Although the WGS allows the identification of plasmid-like contigs, linking them to bacterial species/MAGs is impaired due to separation from the chromosome and differences in genomic features used for binning like oligonucleotide spectrum. One essential advantage of Hi-C metagenomics compared to the WGS is an ability to link the extrachromosomal content to a MAG *via* a chromosome linking signal. Having assessed the plasmid content of Hi-C MAGs (Additional File 9: Supplementary Table 9), we found that the Hi-C MAGs contained a higher number of plasmid contigs than their WGS counterparts (8.8 ± 17.4 vs. 0.94 ± 2.15 across all samples; the cases of highly contaminated WGS-MAGs were not considered here; *p* = 0.005761, Wilcoxon signed rank test with continuity correction, *N* = 18). As a bright example, the *Klebsiella* MAG in the sample IC9 included a plasmid contig of 109,640 bp – representing an almost complete plasmid [according to NCBI nr search, almost perfectly matching a *K. pneumoniae* plasmid previously described for an isolate obtained in Saint Petersburg, Russia (GenBank ID: CP066857.1)] – that was absent in the corresponding WGS-MAG. We further confirmed the circularity of the plasmid by analyzing the assembly graph: the contig ends were overlapping by 55 bp (Additional File 1: Supplementary Figure 5).

### Antibiotic Resistance Analysis

Using an assembly graph based approach implemented in the GraphAMR pipeline, we evaluated the total resistome of each sample (Additional File 10: Supplementary Figure 6). Contrarily to patient A whose semiquantitative AR profiles were remarkably similar between the two time points, the resistome of patient B manifested profound changes in the presence of AR genes (most remarkable were the obtained potential resistance to vancomycin, as shown by occurrence of genes from *van* family, as well as to the tetracyclines suggested by the presence of genes from *tet* family).

We evaluated how Hi-C metagenomics can improve profiling of AR genetic determinants *via* the improved MAG reconstruction. For this analysis, along with the *K. pneumoniae* MAGs, we selected the most abundant Hi-C MAGs (>5%) from each sample (yielding 4–6 MAGs per sample) and identified ARGs in them *via* CARD RGI (see section “Materials and Methods”). Up to 32 AR genes per MAG were detected (including “perfect” and “strict” hits; see Additional File 11: Supplementary Table 10). For patient A, most MAGs carried the *adeF* gene; genes conferring resistance to fluoroquinolones and tetracycline were detected. The *Bacteroides* and *Parabacteroides* additionally showed potential resistance to cephamycin.

For patient B, most MAGs included ARGs related to fluoroquinolones and tetracyclines. The *Bacteroides* MAG at the second time point had genes conferring resistance to cephalosporins (*via* CblA-1 gene specific to *B. uniformis*) – but they were not detected in the *Bacteroides* MAG at the first time point. We checked if it was in fact present at the first point, but in a contig that failed to become binned to a MAG. However, it was not found among the unbinned contigs (Additional File 11: Supplementary Table 10) – likely having failed to be assembled. Possibly, the observation reflects the cefoperazone/sulbactam treatment of the patient (Figure 1).

Following the findings about the higher gene and plasmid contigs counts observed in the Hi-C MAGs compared to their WGS counterparts, we explored the additional value of Hi-C metagenomics in terms of AR. In the example of *Klebsiella*, compared to the WGS-MAGs, the respective Hi-C MAGs included more ARGs – 24–32 vs. 21–22 hits (5–13 vs. 3–4 perfect hits). The Hi-C-specific best ARO (Antibiotic Resistance Ontology) hits (genes, in this context) included, for the patient A, the QnrB1, *dfrA14*, H-NS, QnrS1, OmpA, *msrE* (as opposed to the few WGS-specific ones – QepA2 and TEM-1). For the patient B, the following genes were detected only in the Hi-C MAGs – *mphA*, *dfrA5*, *qacEdelta1*, *sul1*, *sul2*, APH(3′)-VI, NDM-1, QnrS1, *msrE*, *mphE*, BRP(MBL), TEM-1 – while none of the genes were WGS-specific.

Using the Hi-C graph image of resistome, we compared the temporal dynamics of *Klebsiella* resistance potential with the antibiotic regime (see Figure 1). For patient A, the QnrB1 and FosA3 genes were unique to the 1st time point, while the QnrS1 and *msrE* – to the second one. At the level of antibiotic classes, unlike the 1st point, the 2nd time point was characterized by the presence of genes conferring resistance to lincosamide, streptogramin, oxazolidinone and pleuromutilin. Noteworthy, both points were characterized by resistance to carbapenems and aminoglycosides – which is in line with the administered meropenem and amikacin, respectively.

Similar analysis for the *Klebsiella* in patient B microbiome showed that 4 ARGs were baseline-unique (*dfrA5*, *qacEdelta1*, *sul1* and FosA5) and 3 (FosA6, TEM-1 and *catI*) – specific for the 2nd point. No differences between the timepoints were observed at the level of antibiotic classes. Interestingly, while the patient was treated with trimethoprim/sulfamethoxazole, no genes conferring resistance to the drug was identified in the *Klebsiella* MAGs – it is in line with the observation that its relative abundance strongly decreased (from 5.5 to 0.6%) suggesting therapy effectiveness.

We assessed how the Hi-C-mediated linking of plasmids to bacterial chromosomes improved capturing of bacterial resistance profiles. To do it, we calculated the proportion of ARGs located on chromosomal contigs for the *Klebsiella* MAGs (Additional File 12: Supplementary Table 11). For patient A, most ARGs were located on the chromosome (extrachromosomal 8.3 – 12.5% of all hits and 2–3 out of 5 perfect hit genes). On the contrary, for patient B the extrachromosomal proportion of ARGs was considerably higher (34.4–38.7% of all hits and 10/12–13 of perfect hit genes). Considering the fact that WGS-MAGs almost lacked plasmid-like contigs, for patient B, the resistome would have been considerably underestimated without application of the Hi-C data (and inclusion of plasmid contigs to MAG).

Comparative GraphAMR analysis of the resistome of patient B between the two time points suggested the acquired resistance to vancomycin at the second point (sample IC9) (Additional File 10: Supplementary Figure 6) with many ARGs present (*vanA, vanH, vanX, vanR, vanS, vanZ, vanY*). To date, several different types of glycopeptide resistance have been characterized (Arthur et al., 1993); these correspond to specific operons present in the species (Leclercq and Courvalin, 1997). The presence of *vanA* gene suggests the VanA-type resistance, which was the first among the characterized ones and is the most common. This kind of resistance is mediated by transposon Tn1546 or closely related elements. The complete sequence of this ∼10.8 Kbp mobile element was absent in the assembled contigs, and therefore we analyzed the assembly graph neighborhood of ARG matches as reported by GraphAMR to reconstruct the putative structure of VanA operon.

The sequences of genes of interest were located in a tangled (repeat-rich) region on a plasmid and were scattered across 3 edges of the assembly graph (see Additional File 1: Supplementary Figure 7). All 7 genes were found in the correct order, however, the topology of the assembly graph and the observed read coverage of the edges suggest that two different variants of Tn1546 transposon are actually present in the sample: one containing the IS1251 mobile element and another one – without this element (‘C’ and ‘A’ types of Tn1546 transposons as defined in Wardal et al., 2017). Given the short lengths of the corresponding graph edges (2,122, 2,837 and 1,951 bp), it is not surprising that the assembler was unable to resolve these long repeats and join them into a single contig/scaffold. Exploration of the Hi-C contacts between contigs and MAGs showed that the discovered mobile element in the sample IC9 likely belongs to the *Enterococcus faecium* (normalized link weight between these contigs and *E. faecium* MAG was 0.87 ± 0.84; see section “Materials and Methods” for details on normalization).

**FIGURE 7:**
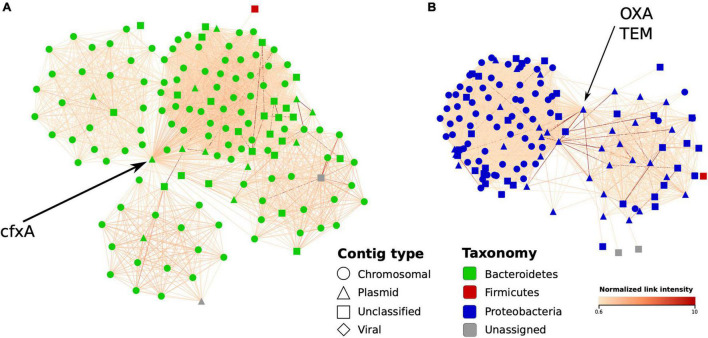
Graph environment of selected antibiotic resistance genes in a Hi-C-based contig network (sample IC6). In each network, vertices correspond to contigs close to a contig containing an AR gene, while the edges – to Hi-C links between the contigs. The layout was constructed using the edge-weighted spring-embedded algorithm. Contig type is shown in shape and bacterial contigs’ taxonomy – in color; edge color denotes intensity of normalized Hi-C link. **(A)** The first graph region of interest: a plasmid-like contig carrying *cfxA* gene is linked to a cluster of contigs classified as belonging to *Bacteroides* genus. **(B)** The second region of interest: a plasmid-like contig carrying OXA and TEM genes linked to a cluster of contigs mostly classified as *Klebsiella*.

Finally, in a complementary MAG-independent network analysis, we evaluated how Hi-C data can be used to detect the bacterial hosts of the ARGs by linking ARG-containing plasmid-like contigs to chromosomal ones. As examples, we used the *Bacteroides cfxA* gene along with the *Klebsiella* OXA and TEM genes – each abundant in sample IC6 and located on plasmid contigs. For each gene, we constructed a network of IC6 contigs linked with Hi-C read pairs around the contig containing the gene (only the contigs > 1,000 bp; the links were normalized as described in section “Materials and Methods”). The obtained environment of the selected ARGs is shown in Figure 7. The *cfxA* gene appeared to be linked with the cluster of contigs classified as Bacteroidetes phylum, specifically as *Bacteroides*, in agreement with the existing knowledge. The OXA and TEM genes were both located on the same contig and linked to Proteobacteria chromosomal contigs classified (using Kraken) as belonging to the *Klebsiella* or *Shigella* genera. Interestingly, this contig was not included into any of the MAGs – showing how information about antibiotic resistance undetected under MAG-based approaches can be identified only by using the contig-level network analysis.

### Linking Prophages to Bacterial Hosts Using Hi-C

Phages are considered to play important roles in microbial ecology. Previous reports showed that Hi-C data can aid in linking them to their bacterial hosts (Marbouty et al., 2017, 2021; Kent et al., 2020). We investigated this approach on our clinical metagenomes. It started with an observation that the Hi-C MAGs list included the items with very low completeness (according to CheckM) but listed among the most abundant MAGs; this effect was observed for both samples of the patient B (IC6 and IC9). A closer examination of IC9 showed that one of such MAGs is composed of 3 contigs classified as crAssphage; their cumulative length was close to the typical genome length for this phage (98 Kbp) suggesting its high completeness. This finding was in agreement with the results of MiCoP showing crAssphage among the most abundant viral sequences (72 and 88% of total viral abundance for IC6 and IC9, respectively). However, investigation of Hi-C links of the crAssphage MAGs did not show link above the established threshold of 0.6 in normalized Hi-C networks for both samples; noteworthy, in the sample IC6, the only suggestive link (0.4) of crAssphage was with the *Bacteroides dorei* MAG. Considering that relative abundance of *B. dorei* did not change much between the two timepoints, one could speculate that at the first point the crAssphage could be presented as a prophage only in a part of the *B. dorei* population, while at the second point – as free phages.

**FIGURE 8:**
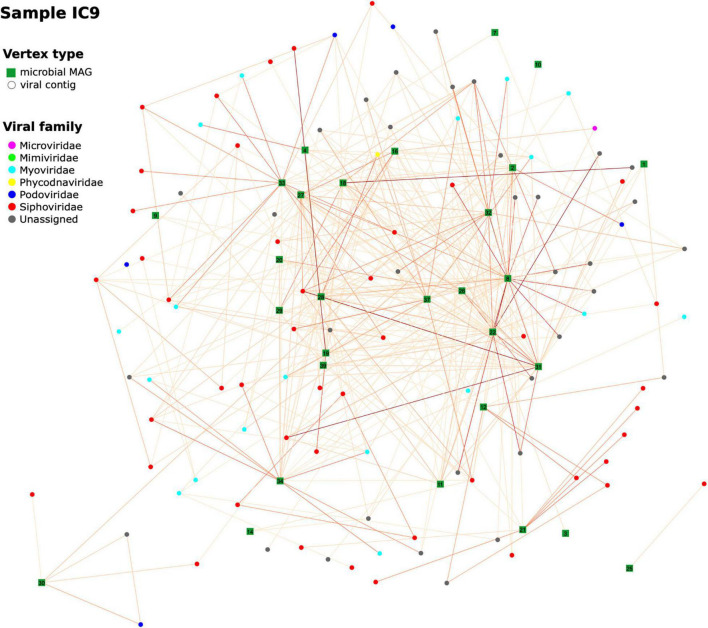
Hi-C-based “virus-bacterial host” networks. The network for the sample IC9 is shown (see Additional File 1: Supplementary Figure 6 for the other samples). Edge-weighted Spring-Embedded algorithm was applied to generate the layout. Vertex shape corresponds to type (MAG or viral contig). The contigs forming each large microbial MAG were combined and shown as a square labeled with MAG ID. Each contig classified as viral was shown as a circle (particularly, if a viral contig had been assigned to a MAG, here it was extracted and drawn as a circle). Vertex color corresponds to viral taxonomy annotation obtained *via* DemoVir. The intensity of edge colors corresponds to the maximum of weight (normalized Hi-C linkage signal) between the contig and all contigs of the MAG. The MAG IDs are as listed in Additional File 3: Supplementary Table 3.

To get a wider perspective on the phages’ hosts, we analyzed viral composition of our samples and evaluated possible “bacteria-virus” associations in each sample, as well as between two time points for each patient. For each sample assembly, viral contigs were identified and annotated (at order or family level). From 1,156 to 1,612 contigs per sample initially defined as viral, the numbers remaining after taxonomic assignment and verification were 333–505; their median length was 3,092–3,233 bp. A network of viral contigs and bacterial MAGs was constructed by considering all Hi-C links between them with normalized weights > 0.6 (Figure 8 and Additional File 1: Supplementary Figure 8). Some viral contigs formed dense clusters with one MAG, while others had strong Hi-C links with several MAGs, thus forming entangled networks. Interestingly, while we detected 107–159 viral contigs per sample each associated with just one MAG, there were many (12–167) contigs per sample that had strong connections to multiple MAGs (Additional File 1: Supplementary Figure 9). Binning algorithms conservatively assigned such viral contigs to at most one MAG, while direct assessment of Hi-C links provided the way to discover all possible hosts of a virus.

Furthermore, some viral contigs have a great number of Hi-C links between each other, possibly reflecting low assembly quality, when the algorithm failed to assemble a single viral sequence and left multiple contigs. Patient B has a sparse network at the first time point characterized by a number of distinct clusters – in contrast to the 2nd time point when the number of links increased considerably to form a dense network. This might reflect changes in microbial ecology linked to intensive therapy.

We additionally explored the taxonomic patterns of “bacteria-virus” associations by estimating the Hi-C links between bacterial MAGs and viral contigs across the samples (Additional File 1: Supplementary Figure 10). Overall, there were 8 viral families associated with bacteria in at least one of the samples; the number of bacterial families detected as being involved in these links was 25. Among the viruses, the most prevalent connections were represented by *Siphoviridae* and *Myoviridae* families (along with the unclassified families from the *Caudovirales* order). In each sample, each of these three viral groups had links with bacterial hosts – most frequently with the members of *Enterobacteriaceae*, *Oscillospiraceae* and *Desulfovibrionaceae* families. On average, the *Siphoviridae* viruses manifested the highest number of links – in agreement with the fact that it was the most abundant family among the viral contigs. These results show how Hi-C signals help to reveal the taxonomic complexity of interactions between phages and their hosts in the human gut.

To compare these findings with a complementary approach, we looked for links between viral contigs and high-quality MAGs using the VirMatcher tool based on multiple criteria (see section “Materials and Methods”). Overall, the number of the discovered associations was 22–76 per sample (Additional File 13: Supplementary Table 12). When compared with the results of our Hi-C analysis, the overlap was 8–30 links confirmed by both methods (in this way, 3.0–6.1% of the Hi-C findings were supported *via* VirMatcher). There could be various reasons for incomplete confirmation. Besides methodological ones like possible false-positive detections of Hi-C network approach and insufficiently complete assembly, the bacteria might lack CRISPR corresponding to a virus but still have alternative methods of defense against it, to name a few – chemical defense, preventing adsorption, restriction–modification and related defenses, and Argonaute proteins (Hampton et al., 2020).

## Discussion

In one of the first Hi-C-aided clinical metagenomic studies presented here, we applied Hi-C metagenomics for deeper exploration of the gut microbiome in chronically critically ill patients. On the example of most abundant opportunists we show how this approach can be beneficial in the clinical context.

Augmentation of WGS sequences with the microbiome-wide chromosome conformation capture data (Hi-C) during binning of contigs resulted in better bacterial genome reconstructions – higher proportion of binned contigs, higher number of quality MAGs and contamination lower by 1–2 orders of magnitude. Furthermore, the novel hicSPAdes algorithm proved to perform better than bin3c, a representative state-of-art Hi-C-based binner. The key difference between hicSPAdes and bin3c is that the latter operates on assembled contigs, while the former uses both contigs and assembly graph. The additional use of topology of the assembly graph improves the binning results as it circumvents the Hi-C data coverage gaps, allows better resolution of repetitive contigs, etc. As a result, the bins obtained by hicSPAdes are more complete and pure. The hicSPAdes is supplemented by the BinSPreader stage that further refines the binning as it allows for assignment of contigs to multiple bins at the same time and splitting the input reads for subsequent reassembly of individual MAGs (this procedure may further improve the contiguity of MAGs due to lesser influence of interspecies repeats).

Comparison of the community structures across the analyzed samples highlighted *Klebsiella pneumoniae* as an omnipresent opportunist which can be used as an example to explore the opportunities of Hi-C metagenomics. This species is a major cause of hospital-acquired infections world-wide including pneumonia, urogenital tract infections (UTIs) and bloodstream infections, especially in immunocompromised individuals, and represents a substantial healthcare burden (Martin and Bachman, 2018). Its morbidity potential is amplified by extensive virulence potential and multi-drug resistance encoded in its open accessory genome, much of which is carried on plasmids. We demonstrated how the consideration of Hi-C data during MAG reconstruction improves the capture of *K. pneumoniae* plasmid content, particularly, the antibiotic resistance genes. It allowed us to detect important virulence factor genes absent in the WGS profiles and, moreover, to identify the inter-individual differences in VF content which was not revealed by conventional metagenomics. Hi-C metagenomics looks especially promising for improving the reconstruction of the mobile genetic elements within genomes that represent problematic regions for assembly and binning. Better reconstruction of MAG improved the accuracy of the downstream comparative genomic analysis; besides the steps described in the manuscripts, it has implications for SNP/indels analysis, phylogenetics and so on. The approach can be applied to other opportunist taxa actively involved in HGT and notorious for their virulent and multidrug-resistant members like *Enterococcus* and *Escherichia*.

There were interesting observations among the taxonomic compositions that might have clinical significance for the critically ill patients. In 3 of 4 samples, we detected a high abundance of *Cloacibacillus* – *C. porcorum* or related – an amino acid degrading microorganism capable of using mucin as a sole carbon source (Looft et al., 2013). The species is a potential gut beneficiary of chronic critical illness linked to muscle loss (cachexia) and malabsorption. Particularly, in patient B, at the 2nd time point it might have replaced another – commensal – mucose-dwelling species *Akkermansia muciniphila* abundant at 1st point. Three of the 4 samples included an abundant MAG classified as OEMR01, a member of the *Erysipelotrichaceae* family. The links of *Erysipelotrichaceae* members to host health are yet to be elucidated; they appear to be highly immunogenic and can thrive after treatment with broad-spectrum antibiotics (Zhao et al., 2013; Kaakoush, 2015). According to our previous 16S rRNA sequencing study, the *Erysipelotrichaceae* was enriched in the gut microbiome of CCI patients compared to the patients in acute critical state (Chernevskaya et al., 2020).

In a clade-specific marker analysis, we identified fungal sequences in each sample. Fungi can represent significant health risks for critically ill patients. As the coverage was low, we did not recover fungal MAGs, not to mention the sufficient Hi-C signal (additional experimental enrichment of the fungal fraction would be required). We anticipate that in such cases, involvement of Hi-C metagenomics to bin fungal genomes consisting of multiple chromosomes will be indispensable. One of the interesting results of the study is the dominance of *Enterocytozoon bieneusi* in the composition of fungiome, an obligate intracellular parasite infecting intestinal cells agent of intestinal microsporidiosis that can manifest as diarrhea (Weiss and Becnel, 2014). The condition can be life-threatening in immunocompromised patients, particularly in the chronically critically ill group.

Overall, the Hi-C-assisted MAG reconstruction performed well for the sufficiently covered microbial genomes. Recovery of low-abundant microorganisms would require higher targeted sequencing coverage. Noteworthy, as the ICU patients often manifest low alpha-diversity (intestinal domination of a single species as an extreme case), the chance of obtaining good-quality genome reconstructions is higher than for healthy subjects hosting more diverse communities. We found that not all high-covered taxa produced good-quality MAGs. This might be related to the variability of GC content. One of the possible experimental solutions would be to use multiple restriction enzymes during the Hi-C library preparation based on the sequence analysis of major expected genomes (Magnitov et al., 2020).

Completeness, the central measure of prokaryotic MAG quality, is commonly based on evaluation of chromosomal single-copy core genes and thus does not take plasmids into account (Parks et al., 2015). Meanwhile, their gene content can drastically affect the bacterial host phenotype, which is especially important for the clinically relevant gut microorganisms. Hi-C metagenomics renders the plasmid content of species detectable and allows to come up with a concept of “plasmid completeness” of microbial genomes reconstructed from metagenomes.

A crucial domain of microbial phenotypes in the clinical context is their drug resistance. The Hi-C data allowed improving resistome profiling – as seen even at the level of MAGs. Although chronic critical illness following severe non-traumatic brain damage was common in these patients, they showed different clinical – as well as microbiome – trajectories. The non-survived patient A had been given antibiotics for a long time prior to the first time point and her therapy was quite constant between the timepoints. At the level of her microbiome, it was reflected by similar species-level composition at the two points and resistome – the latter being comparable by both total ARG relative abundance and presence patterns. On the other hand, the microbiome of patient B (ultimately recovered) who started antibiotic administration at the 1st time point was characterized by strong changes in taxonomic composition with quantitative and qualitative alterations of the resistome. As the pilot sample size was small and the set of prescribed antibiotics varied between the patients, we cannot claim significant effects of the therapy on gut resistome. Various administered drugs showed diverse patterns. For example, for patient A, at baseline, potential resistance to some discontinued drugs increased (possibly reflecting the recovery of a low-abundant resistant population), while for some it decreased (might be removed by negative selection).

The results of ARG prediction even in average-complexity metagenomes (such as the human gut) could be significantly affected by fragmented assemblies. We demonstrated that the use of assembly graph-based approaches is far superior in terms of recovery of more complete ARG sequences even from fragmented metagenome assemblies. Specialized pipelines such as GraphAMR could be used to improve the current approaches of ARG prediction of metagenomic assemblies. Hi-C data could be used to further validate and confirm the results obtained. One essential problem here is that during the assembly, an ARG sequence present in multiple species is likely to be included – flattened into a part of a single contig – into a single MAG. Our results suggest that for deeper resistome profiling using Hi-C, it is promising to operate directly on assembly graphs – prior to formation of contigs and binning of them into MAGs.

Another entity in the gut microbiome that is highlighted by the Hi-C metagenomics are phages. Phages are considered to contribute considerably to the regulation of gut microbial communities (Sutton and Hill, 2019). Identification of their bacterial hosts can help elucidate the precise mechanisms of their contribution. One of the most studied phage families are crAss-like phages; crAssphage’s host has been identified to be *Bacteroides intestinalis* (Shkoporov et al., 2018). Previously, it was shown for the same population as the present study (Russian) that the crAssphage reads can represent as much as 24% of the stool metagenome (Yarygin et al., 2017). Recent study in healthy subjects showed using the Hi-C metagenomics how phages of this group can be linked to various species within the *Bacteroides* genus (Marbouty et al., 2021). In our study, although we discovered high levels of crAssphage persisting in one of the patients between the time points, there were no strong links to any bacterial MAGs. The fact that there was a slight contact to *Bacteroides dorei* at the first point only suggests underlying dynamics of proportions between prophages and free phages. After expanding our analysis from this providential occurrence to a global analysis of “virus-bacterial host” network using Hi-C, we discovered the presence of viral contig hubs linked to multiple hosts. Although this could partially be due to misassemblies, such results may hint at possible promiscuity of phages. This can have implications for transmission of ARGs and virulence factors determinants across diverse gut species in immunocompromised patients (however, we have not detected ARGs in viral contigs in our data).

One of the challenges in the present study was to discern signal from noise in Hi-C metagenomic data. It is possible to determine a proper threshold *via* additional experiments on defined bacterial consortia with plasmids, preferably those of high diversity comparable to human gut. In the absence of such opportunities, we determined the threshold by assessing the inter-intra-MAG links distributions. In our case, the separation of distributions choice was visually similar between the 4 samples and the false discovery rate of detecting an inter-MAG was quite low (from 0.0082 for IC6 sample to 0.0351 for IC5, see Supplementary Figure 2B). The specific threshold value is likely to vary for new datasets or under experimental protocol modifications. Therefore, it is recommended to evaluate such distributions for each particular dataset.

Another limitation is a small sample size – that did not provide an opportunity to assess statistical significance in some analyses. However, our study did not set an objective of comparing the two patients with each other, but we had rather initially selected the most interesting representative examples of CCI patients in order to illustrate the broad possibilities of the clinical Hi-C metagenomics as a method. In connection to the specific clinical group (the critically ill patients), the analysis of statistical power and sufficient sample size face a heterogeneity challenge: in the ICU, the treatment (including the choice of antibiotics) based on individual clinical status and dynamics strongly varies across the subjects. Therefore, the inter-subject variability of clinical factors is much higher than for typical major diseases linked to microbiome composition in metagenome-wide association studies (like inflammatory bowel disease or type 2 diabetes). It follows that for a strict statistical analysis – with proper adjustment for the confounding factors – and considering the inherent high dimensionality and multimodality of microbiome data, the required sample size might be very large. In our previous survey of CCI patients’ gut microbiome (Chernevskaya et al., 2021), even among 44 patients at the group level we did not observe a prevalent antibiotic therapy pattern – the individual treatments were highly variable. Nevertheless, despite different diagnoses, the chronically critically ill patients convergently acquire the same features of the clinical course, with profound changes in the gut microbiome. Thereby, the dataset in this study allowed us to demonstrate how Hi-C metagenomics can be expedient in the context of clinical metagenomics. The technique is yet to become affordable for wide application. However, further analysis of larger cohorts might provide the basis for developing simpler targeted and cost-effective methods like 3-C for specific clinical aims.

In the ICU microbiome research, the technique can be readily applicable to analysis of other body sites, as well as for hospital surfaces – that can serve as media of pathogens transmission. It is also relevant to the COVID-19 pandemics: it is estimated that not less than ∼7% of COVID-19 patients develop bacterial co-infection and most lethal outcomes in the ICU are ultimately determined by this factor. As the gut microbiome is an important reservoir of opportunistic infectious agents causing such invasions, its virulence and drug resistance potential should be explored in detail.

## Conclusion

Hi-C metagenomics is a promising tool for analyzing clinical microbiome samples. Compared to conventional metagenomics, it provides reconstructed microbial genomes of higher completeness and lower contamination. In the context of critical care, the method coupled with specialized algorithms improves the precision of profiling antibiotic resistance and virulence potential of opportunist gut taxa, as well as the tracking of mobile genetic elements dynamics. The findings can help optimize the treatment schemes and understand mechanisms of pathogenesis in the ICU.

## Data Availability Statement

The WGS and Hi-C sequencing data are available in the European Nucleotide Archive (ENA) repository under the accession number PRJNA718195. The data processing scripts are available at https://bitbucket.org/ibg_super/hicicuscripts/.

## Ethics Statement

The studies involving human participants were reviewed and approved by the Local Ethics Committee of Federal Research and Clinical Center of Intensive Care Medicine and Rehabilitology. Formal consent for participation in this study was obtained from the legal representative of each patient.

## Author Contributions

AT, SU, SR, and EC conceived the study design, experiments and analyses. EC and NB managed sample collection. PV supervised by SU performed sample preparation. VI, AI, IT, VU, and DS developed the software and analyzed the data. VI, AT, and AK interpreted the analyses. AT, SU, AK, and VU coordinated the project. VI, AT, EC, AK, and AI wrote the manuscript with critical revision performed by SU, SR, VU, NB, and PV. All authors read and approved the final manuscript.

## Conflict of Interest

AT was an employee of Knomics LLC and Atlas Biomed Group companies at the time of the study. Sequencing was performed by Knomics LLC. The companies were not involved in the study design, collection, analysis, interpretation of data, the writing of this article or the decision to submit it for publication. The remaining authors declare that the research was conducted in the absence of any commercial or financial relationships that could be construed as a potential conflict of interest.

## Publisher’s Note

All claims expressed in this article are solely those of the authors and do not necessarily represent those of their affiliated organizations, or those of the publisher, the editors and the reviewers. Any product that may be evaluated in this article, or claim that may be made by its manufacturer, is not guaranteed or endorsed by the publisher.

## Footnotes

1
https://cab.spbu.ru/software/hicspades/

2
https://CRAN.R-project.org/package=packcircles

3
https://card.mcmaster.ca/analyze/rgi

4
https://github.com/dyxstat/HiCzin

5
https://github.com/feargalr/Demovir

6
https://bitbucket.org/MAVERICLab/virmatcher

## Abbreviations

ARG: antibiotic resistance gene
CCI: chronically critically ill
HGT: horizontal gene transfer
ICU: intensive care unit
MAG: metagenome-assembled genome
SCG: single-copy core genes
UTIs: urogenital tract infections
VF: virulence factors.
