# Supplementary material for: Hi-C Metagenomics in the ICU: Exploring Clinically Relevant Features of Gut Microbiome in Chronically Critically Ill Patients
Source: Front Microbiol. 2022 Feb 3;12:770323. doi: 10.3389/fmicb.2021.770323 (PMC8851603; doi:10.3389/fmicb.2021.770323)
Supplement: Supplementary file 1 [file Data_Sheet_1.zip › Additional file 1.pdf]

Supplementary materials

Supplementary text **1**
Software versions 1
Command lines 2
Supplementary figures **2**
Supplementary tables **12**

Supplementary text

Software versions

- anvi'o v.7
- bbmerge v.37.62
- bin3c v.0.1.1
- CheckM v.1.0.13
- Cytoscape v.3.7.2
- DemoVir v.1 (downloaded from https://github.com/feargalr/Demovir in February 2021)
- FastTree v.2.1.10
- GraphAMR revision 9a22f168580da24019f234a9.
- GTDB-Tk v.0.3.2
- hicSPAdes binning module v.0.1 (available from https://cab.spbu.ru/software/hicspades/)
- HiCzin (downloaded from https://github.com/dyxstat/HiCzin in September 2021)
- InSilicoSeq v.1.5.2
- Kraken v.2.1.1
- MetaBat 2 v. 2.12.1
- MetaPhlAn2 v.2.7.7
- MiCoP (downloaded from https://github.com/smangul1/MiCoP in February 2020)
- packcircles R package v. 0.3.4
- PHASTER (Web-service; accessed at https://phaster.ca/ in March 2021)
- PlasFlow v.1.1.0
- Prodigal v.2.6.3
- qc3C v.0.2.6.3
- RGI (Web-service; accessed at https://card.mcmaster.ca/analyze/rgi in December 2020)
- Sim3C v.0.2
- SPADES v.3.15
- Tablet v.1.19.09.03
- UpsetR package v.1.4.0
- VFanalyzer (Web-service; accessed at http://www.mgc.ac.cn/cgi-bin/VFs/v5/main.cgi?func=VFanalyzer in December 2020)

- ViralVerify (pre-release version, downloaded from https://github.com/ablab/viralVerify in December 2020)
- VirMatcher (downloaded from https://bitbucket.org/MAVERICLab/virmatcher in November 2021)

Command lines

- hicSPADES:
  hicspades-binner <graph.gfa> <hic_reads.yaml> <output>
- GraphAMR:
  nextflow run ablab/graphamr -profile conda --graph '*.gfa' --db 'card_AA'

Supplementary figures

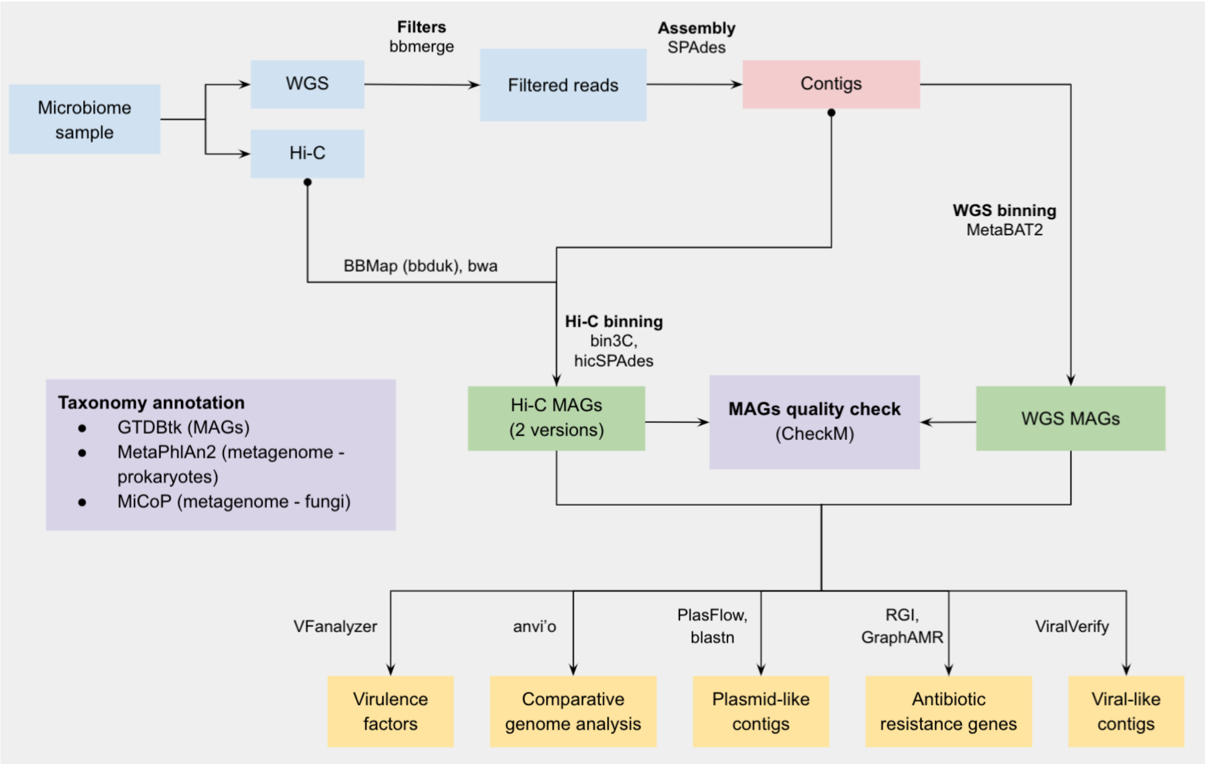

Figure S1 - WGS and Hi-C data analysis workflow.

A)

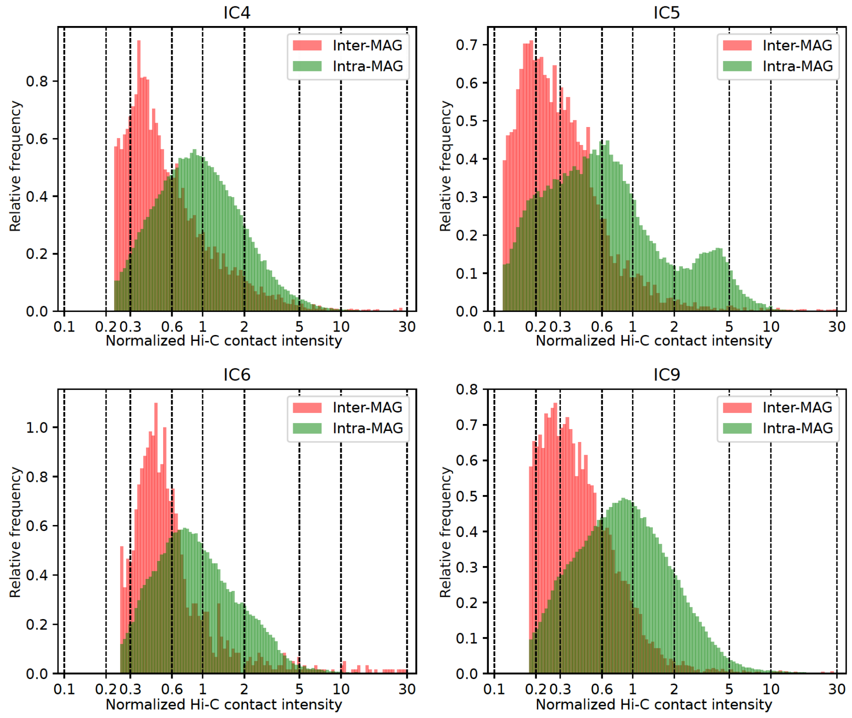

B)

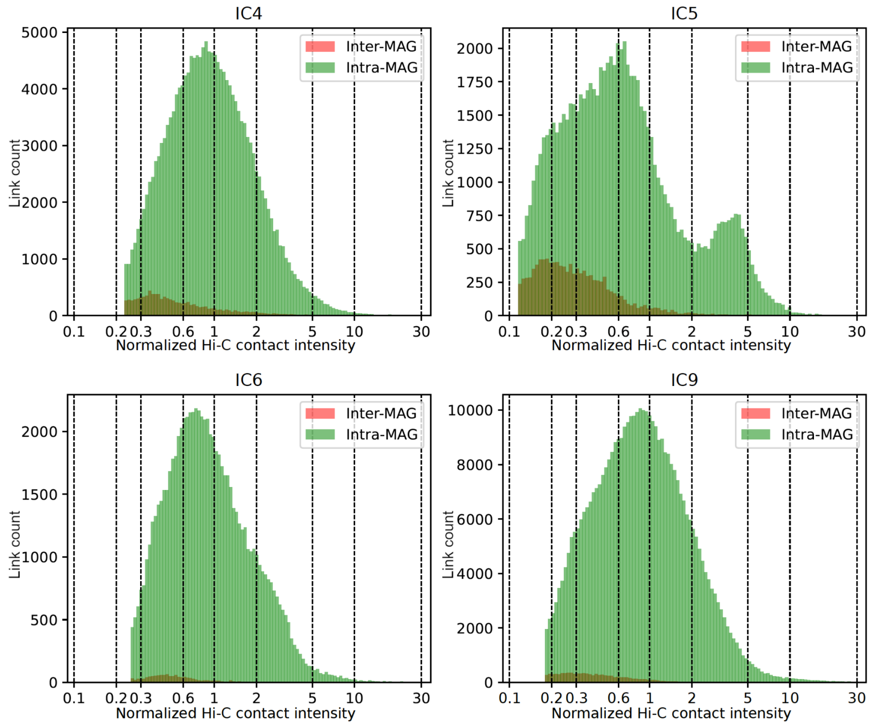

Figure S2 - Sample-wise distribution of normalized Hi-C links intensity between the contigs belonging to the same high-quality MAG (“intra-MAG”) and those belonging to different MAGs (“inter-MAG”). The horizontal axis is visualized in log-scale. For visual appearance, several Hi-C values have been highlighted with vertical dash lines. Among

them, 0.6 was selected as visually optimal “noise-signal” threshold. A) With distribution normalization to 1. B) Without distribution normalization (link counts).

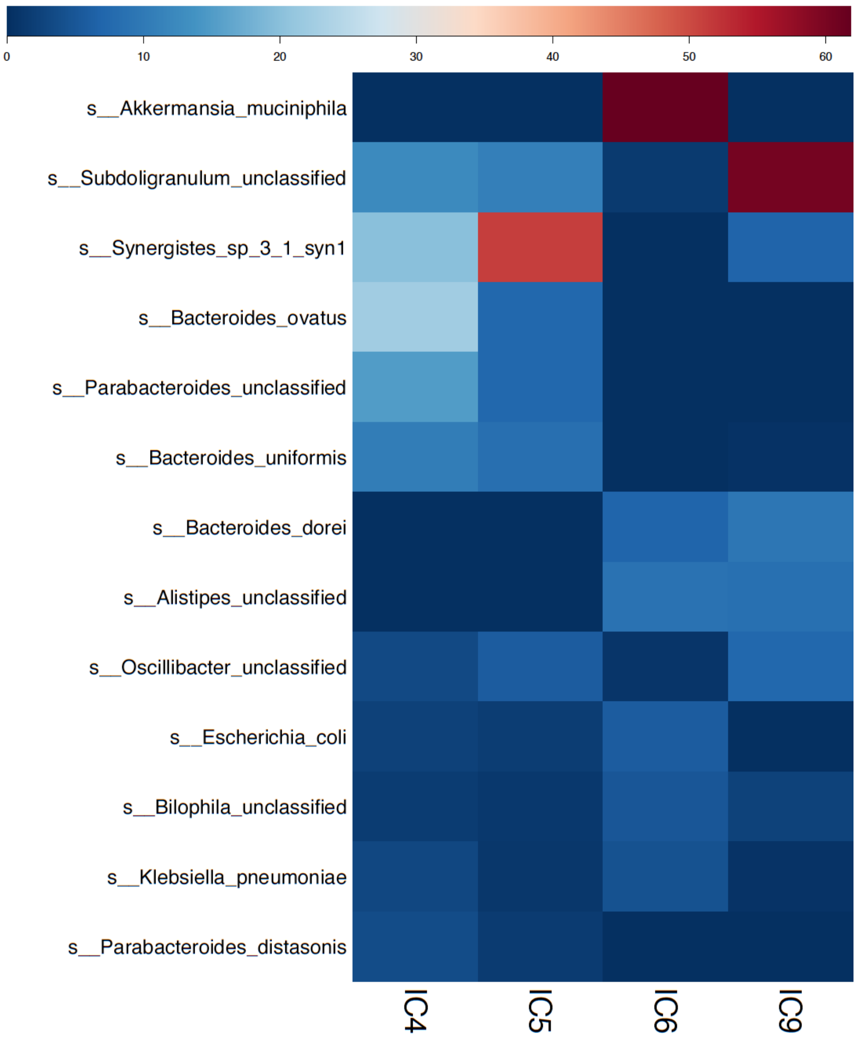

Figure S3 - Taxonomic composition of gut microbiome in CCI patients: clade-specific marker gene analysis (MetaPhLan2; genus level). Each cell reflects relative abundance of a bacterial genus (in percent).

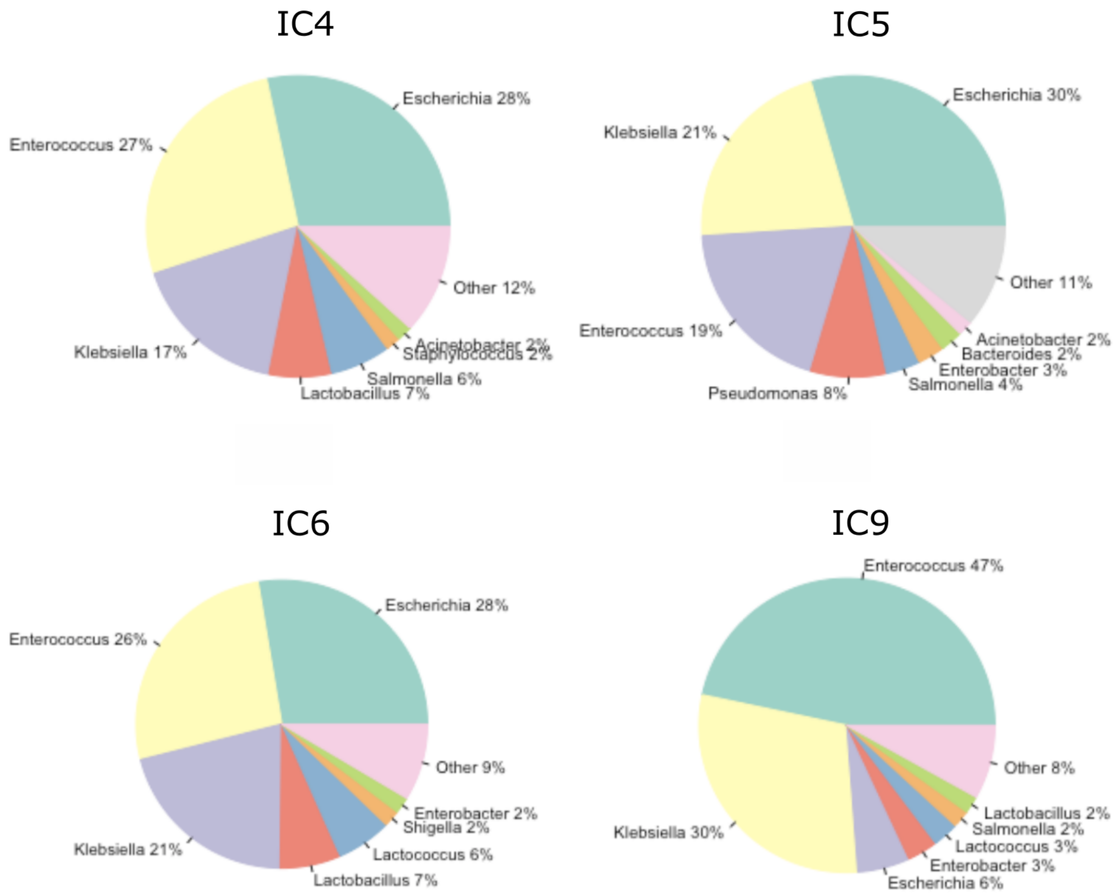

Figure S4 - Taxonomic annotation of potentially plasmid contigs (for each sample).

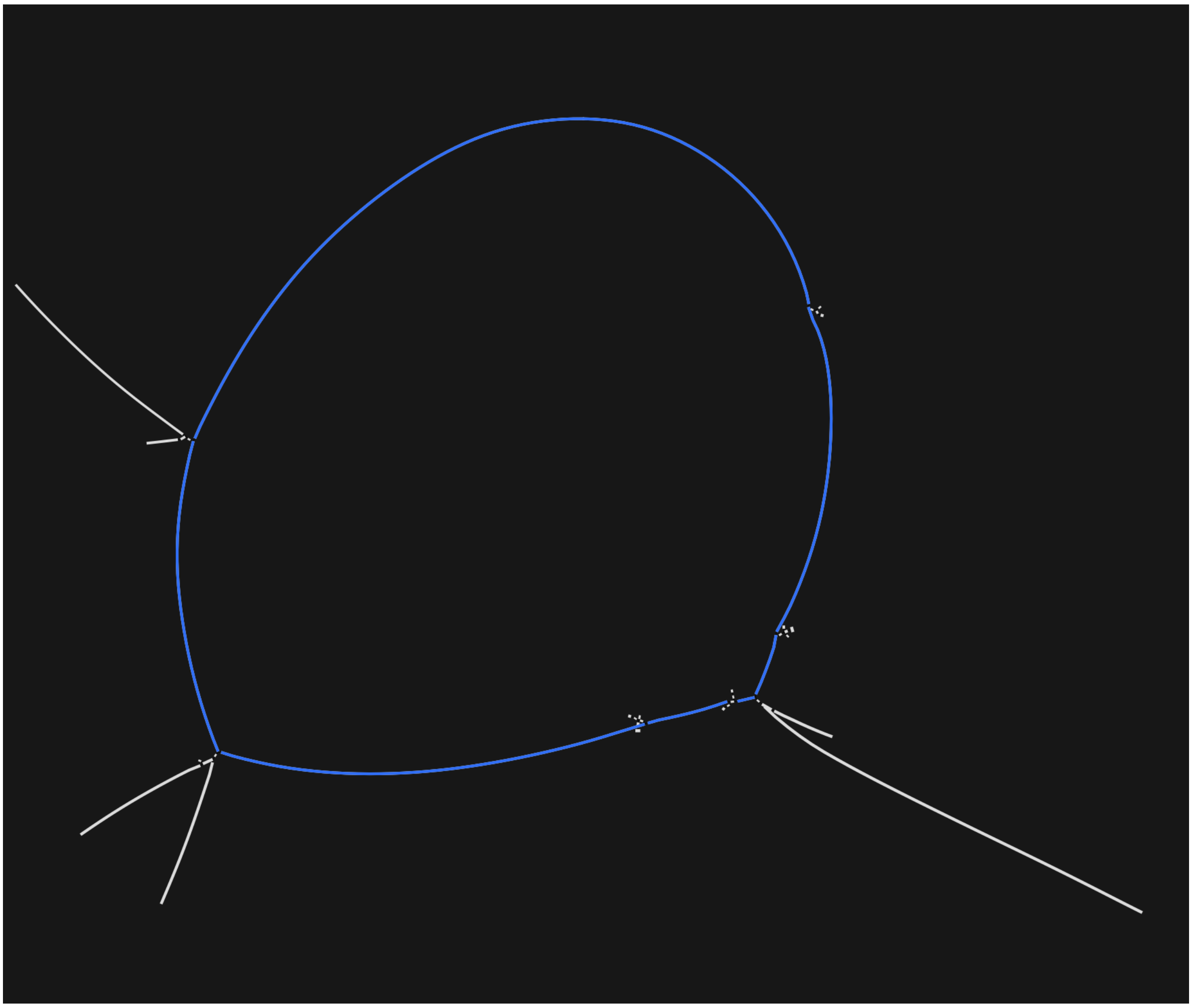

Figure S5 - Fragment of the IC9 assembly graph illustrating the circularity of *Klebsiella plasmid* contig. Assembly graph nodes are shown as lines scaled to their length; the nodes corresponding to the plasmid contig are shown in blue, while the grey lines correspond to flanking graph edges. Visualization was performed in Bandage (Wick et al, 2015). Plasmid length is 109,650 bp with 100% IDY match to CP066857.1.

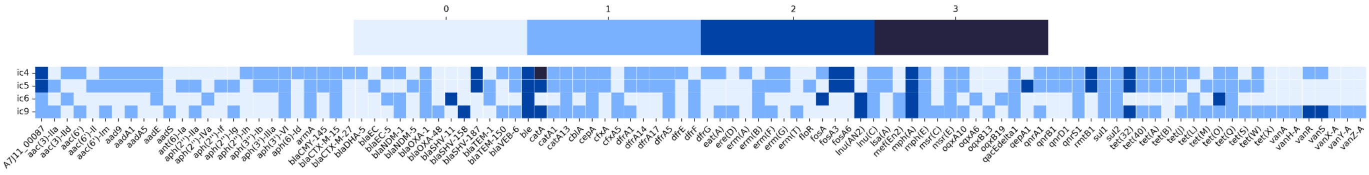

Figure S6 - Resistome diversity of the samples. The intensity of color denotes the number of unique (at 95% AA IDY) matches to a particular ARG gene family as reported by Abricate (https://github.com/tseemann/abricate).

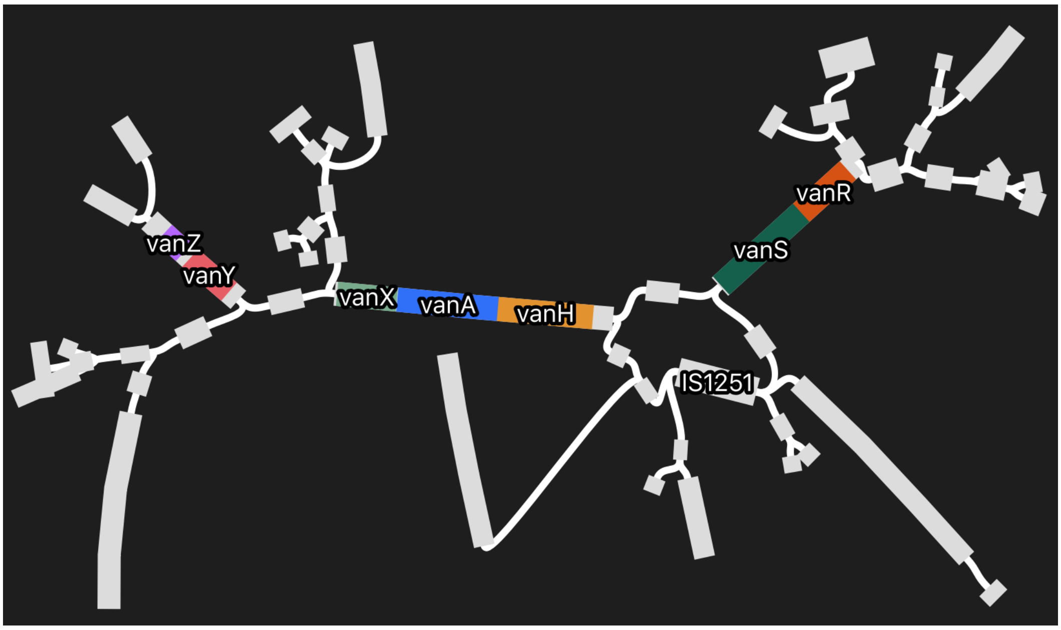

Figure S7 – Part of assembly graph containing VanA operon conferring resistance to vancomycin (sample IC9). Different genes are scattered across 3 graph edges and are color-coded. In addition, the IS1251 sequence is outlined suggesting putative 1.5 Kbp insertion between the *vanH* and *vanS* genes in one variant of VanA operon. The length of *vanZ*+*vanY* contained edge (left) is 2,122 bp, *vanX*+*vanA*+*vanH* (middle) edge is 2,837 bp, and *vanS*+*vanR* edge (right) is 1,951 bp.

A)

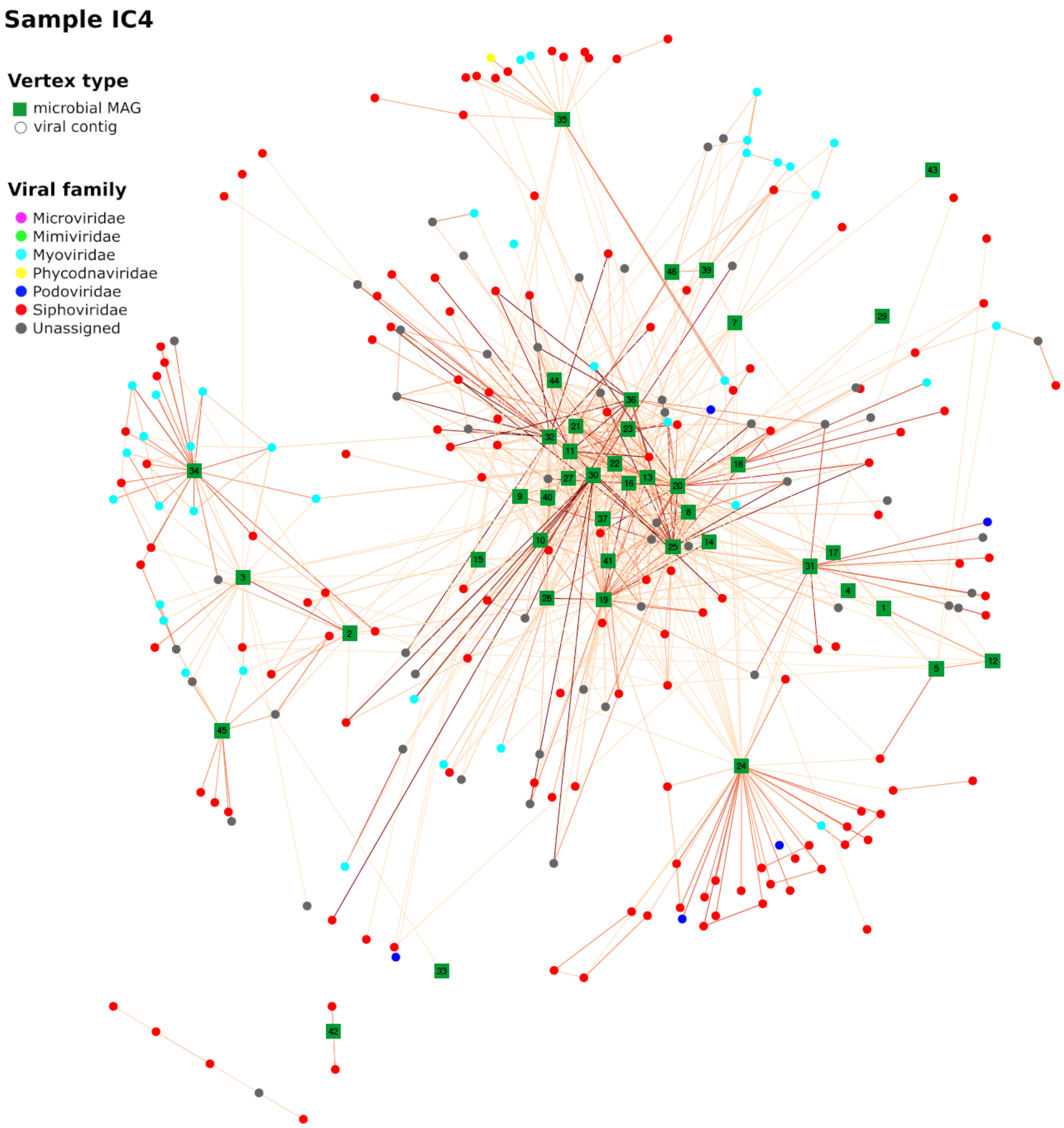

Sample IC4
Vertex type
microbial MAG
viral contig
Viral family
Microviridae
Mimiviridae
Myoviridae
Phycodnaviridae
Podoviridae
Siphoviridae
Unassigned

B)

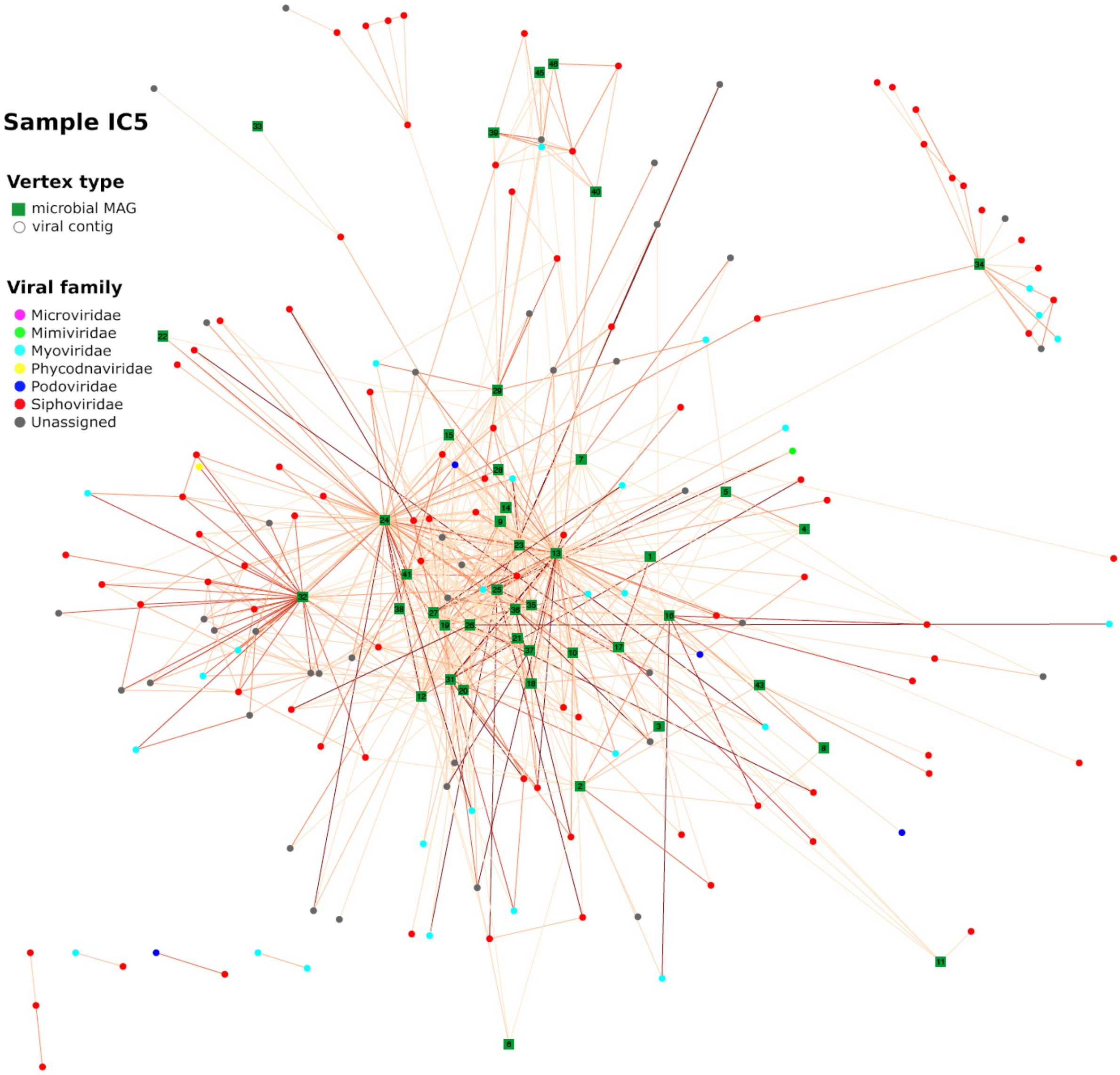
Sample IC5
Vertex type
microbial MAG
viral contig
Viral family
Microviridae
Mimiviridae
Myoviridae
Phycodnaviridae
Podoviridae
Siphoviridae
Unassigned

C)

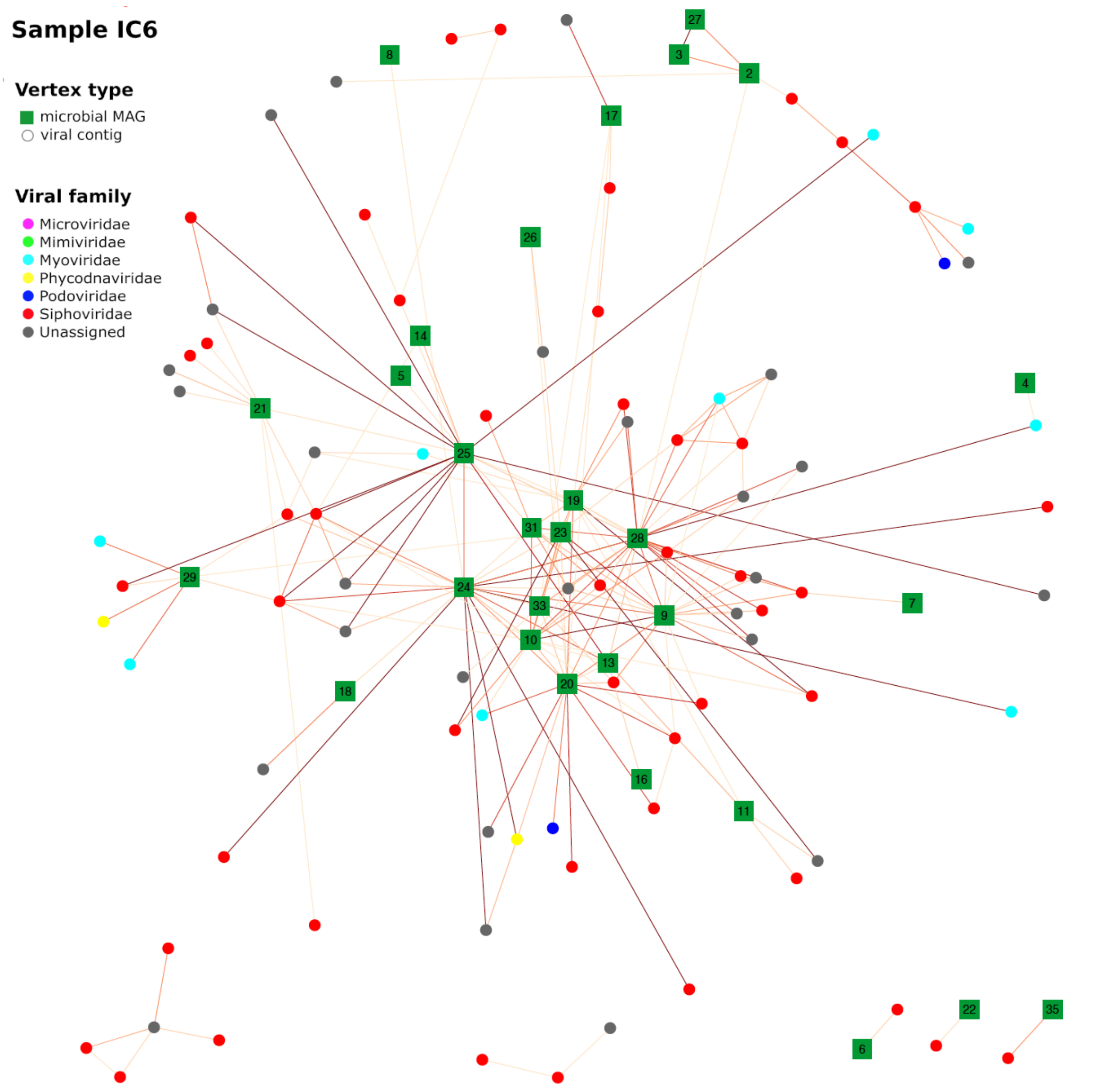

Figure S8 - Hi-C-based "virus-bacterial host" networks for the samples IC4, IC5 and IC6. Constructed similarly to Figure 8.

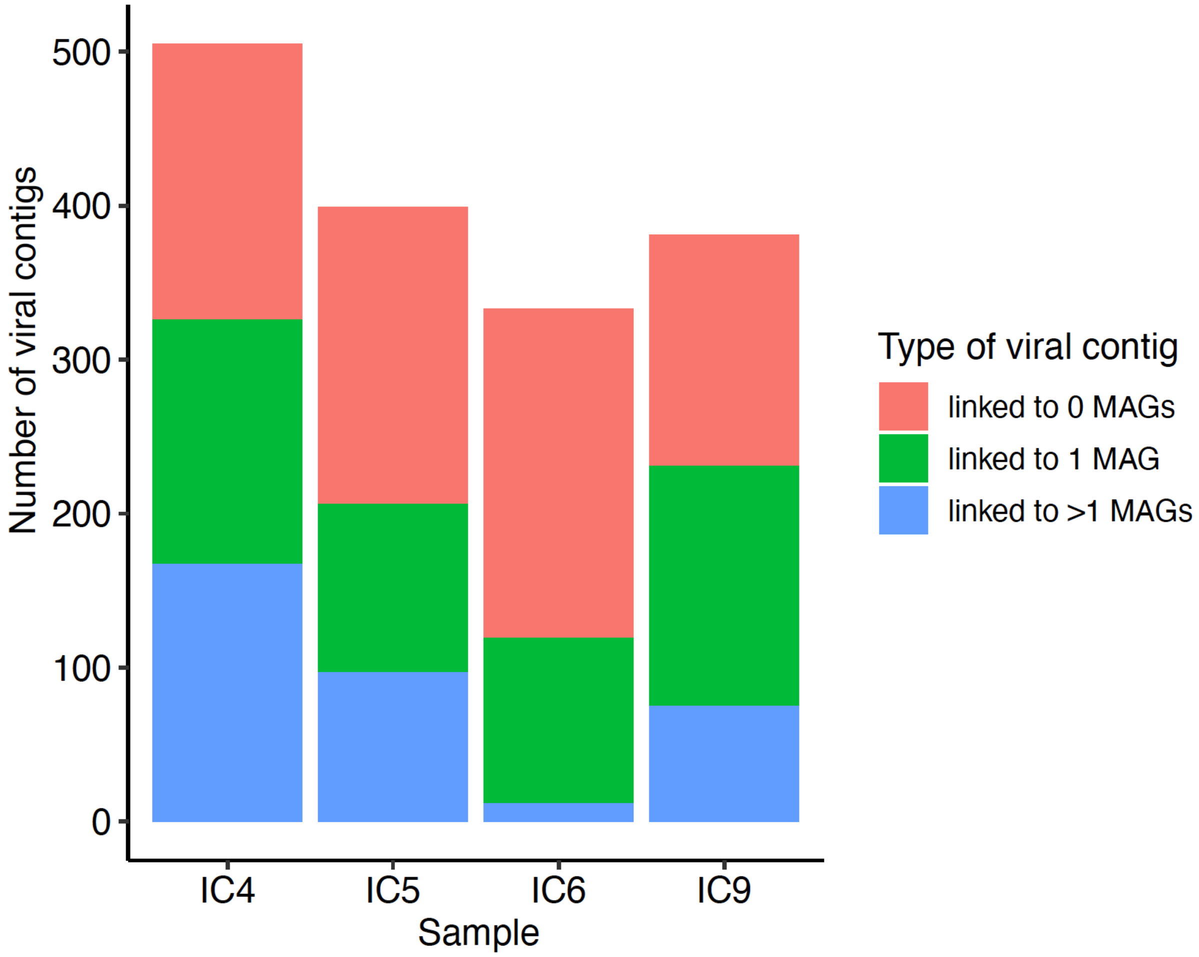

Figure S9 - Statistics on "bacteria-virus" interactions. For each sample, the number of its viral contigs is shown divided according to the number of bacteria (high-quality MAGs) they are associated with. The analysis is based on normalized Hi-C contact network; the threshold for linkage between MAG and contig is 0.6.

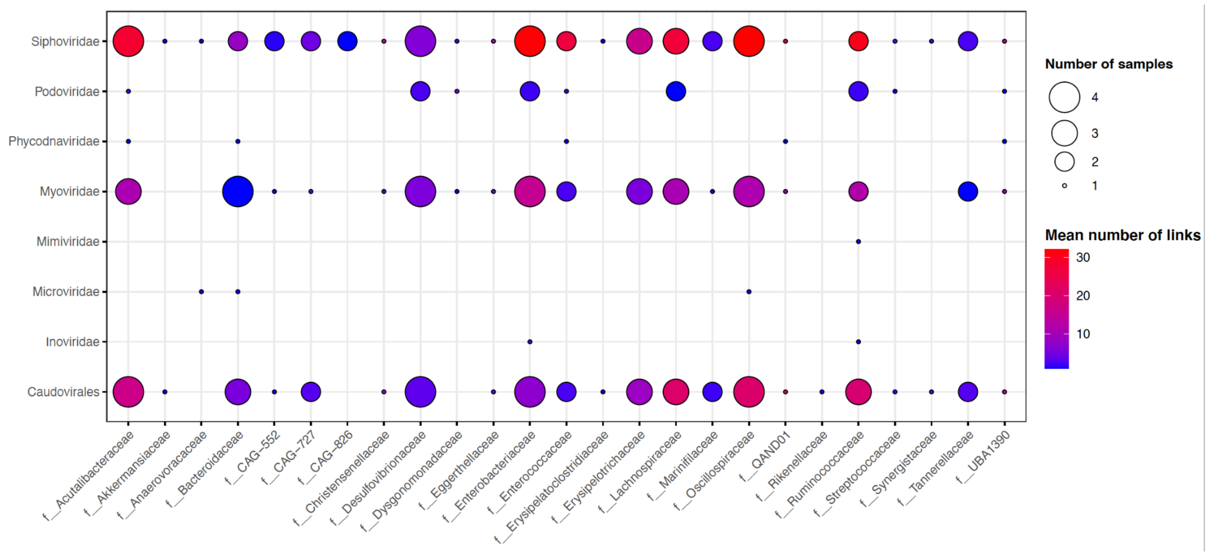

Figure S10 - Taxonomy of “bacteria-virus” associations. The bubbleplot shows the sample-wise prevalence and abundance of links between viral (rows) and bacterial (columns) taxa derived from Hi-C data. The number of associations was evaluated by counting viral contigs linked to each high-quality bacterial MAGs in normalized Hi-C network for each sample, aggregating the links by viral and bacterial families and averaging across the samples (visualized as circle colour). The number of samples in which a link between viral and bacterial family was detected was denoted as circle size. Only the taxa involved in a link in at least one sample are shown. Only the links with normalized weight >0.6 have been considered (see Materials and Methods). The row label “Caudovirales” refers to the unclassified families from the respective order.

Supplementary tables

Table S1 - Patient data.

<table>
<tr><th></th><th>Patient A
(samples 4 - 5)</th><th>Patient B
(samples 6 - 9)</th></tr>
<tr><td>Age/Gender</td><td>75 / F</td><td>74 / M</td></tr>
<tr><td>Diagnosis</td><td>intracerebral hemorrhage</td><td>ischemic stroke</td></tr>
<tr><td>Length of stay in ICU, w</td><td>8</td><td>6</td></tr>
<tr><td>Support</td><td colspan="2">Mechanical ventilation,
enteral tube feeding (high calorie, low-residue)</td></tr>
<tr><td>Timepoint 1</td><td colspan="2">suspected bacterial infection
pathogens in trachea (cultivation)
pneumonia signs from CT Scan</td></tr>
<tr><td>Timepoint 2</td><td>+1 week, negative clinical dynamics</td><td>+2 weeks, positive clinical dynamics</td></tr>
</table>

Additional file 2: Table S2 - Concentrations of selected gut taxa in patients' stool samples measured using real-time qPCR (in CFU/ml).

Additional file 3: Table S3 - Composition of fungal community on species level (according to MiCoP, in percentage from total fungal abundance).

Additional file 4: Table S4 - Summary statistics of libraries, assemblies and binnings.

Additional file 5: Table S5 - Coverage, quality and taxonomic assignment of Hi-C MAGs produced using bin3c. A-D) For each sample. E-F) For each patient-wise cross-assembly of 2 samples. For the E and F, color fill shows how MAG quality changed between the timepoints: dark green - improved MAG completeness, green - taxonomically new MAG, red - decreased MAG completeness, white - MAGs whose completeness is approximately the same as the highest across the two samples.

Additional file 6: Table S6 - Quality and taxonomic assignment of WGS MAGs produced using MetaBat2 (CheckM statistics).

Additional file 7: Table S7 - Coverage, quality and taxonomic assignment of Hi-C MAGs produced using hicSPAdes.

Additional file 8: Table S8 - Distribution of virulence-related genes in *K. pneumoniae* MAGs. The VFanalyzer statistics are provided for each WGS and Hi-C MAG. The rows corresponding to subject-specific VF genes are filled with green. The cells showing the genes detected in the WGS MAGs but not their Hi-C counterparts are filled with yellow.

Additional file 9: Table S9 - WGS and Hi-C MAGs compared by their plasmid content. For each WGS MAG, the results of its bin3c-produced Hi-C counterpart is provided. The results include the number of plasmid-like contigs and their taxonomic annotation at genus level. Only the major MAGs are shown (relative abundance > 5%).

Additional file 10: Table S10 - Antibiotic resistance potential of Hi-C MAGs. For each sample, the information is provided for the *K. pneumoniae* MAG, the highly-abundant MAGs as well as for the unbinned contigs in total. For each ARG, there is information about its best Antibiotic Resistance Ontology (ARO) hit, best identities, model type, drug class, resistance mechanism AMR gene family and percentage length of the reference sequence. Specifically for each *K. pneumoniae* Hi-C MAG, the respective information about its WGS counterpart is provided on the right for comparison.

Additional file 11: Table S11 - Antibiotic resistance potential of *K. pneumoniae* Hi-C

MAGs based solely on the contigs predicted to be chromosomal. The information is provided in the same format as in the previous table.

Additional file 12: Table S12 - VirMatcher results for linking viral contigs and high-quality bacterial MAGs. For each sample, all contigs identified as being viral via ViralVerify were input. The table contains only the associations with the VirMatcher final scores ≥3 and for the high-quality MAGs only. The columns show information about the viral contig ID, identified host ID and detailed information about hits for each sub-method (CRISPR-spacers, integrated prophage, tRNA genes, WIsH k-mer signatures) along with the final score.
